# Discovery of potential inhibitors against New Delhi metallo-β-lactamase-1 from natural compounds: in silico-based methods

**DOI:** 10.1038/s41598-021-82009-6

**Published:** 2021-01-27

**Authors:** Azhar Salari-jazi, Karim Mahnam, Parisa Sadeghi, Mohamad Sadegh Damavandi, Jamshid Faghri

**Affiliations:** 1 Department of Microbiology, School of Medicine, Isfahan University of Medical Sciences, Isfahan, Iran; 2 Biology Department, Faculty of Sciences, Shehrekord University, Shahrekord, Iran

**Keywords:** Computational biology and bioinformatics, Predictive medicine, Statistical methods, Virtual drug screening, Antimicrobials, Clinical microbiology

## Abstract

New Delhi metallo-β-lactamase variants and different types of metallo-β-lactamases have attracted enormous consideration for hydrolyzing almost all β-lactam antibiotics, which leads to multi drug resistance bacteria. Metallo-β-lactamases genes have disseminated in hospitals and all parts of the world and became a public health concern. There is no inhibitor for New Delhi metallo-β-lactamase-1 and other metallo-β-lactamases classes, so metallo-β-lactamases inhibitor drugs became an urgent need. In this study, multi-steps virtual screening was done over the NPASS database with 35,032 natural compounds. At first Captopril was extracted from 4EXS PDB code and use as a template for the first structural screening and 500 compounds obtained as hit compounds by molecular docking. Then the best ligand, i.e. NPC120633 was used as templet and 800 similar compounds were obtained. As a final point, ten compounds i.e. NPC171932, NPC100251, NPC18185, NPC98583, NPC112380, NPC471403, NPC471404, NPC472454, NPC473010 and NPC300657 had proper docking scores, and a 50 ns molecular dynamics simulation was performed for calculation binding free energy of each compound with New Delhi metallo-β-lactamase. Protein sequence alignment, 3D conformational alignment, pharmacophore modeling on all New Delhi metallo-β-lactamase variants and all types of metallo-β-lactamases were done. Quantum chemical perspective based on the fragment molecular orbital (FMO) method was performed to discover conserved and crucial residues in the catalytic activity of metallo-β-lactamases. These residues had similar 3D coordinates of spatial location in the 3D conformational alignment. So it is posibble that all types of metallo-β-lactamases can inhibit by these ten compounds. Therefore, these compounds were proper to mostly inhibit all metallo-β-lactamases in experimental studies.

## Introduction

Multi-drug resistance has become a significant threat to global health, and it appears that discovery of new class antibiotics is in an uttermost need for humankind^1^. Globally, beta-lactam antibiotics have been extensively prescribed to treat bacterial infections. The considerable rise of antibiotic resistance in bacteria emanates from excessive use of beta-lactam antibiotics^2^. Beta-lactamases in bacteria are the most common mechanism to hydrolyze beta-lactam antibiotics and their resistance. β-Lactamase enzymes are a bacterial defense mechanism to hydrolyze amide bond in beta-lactam antibiotics that inactivate them. According to the Ambler classification, beta-lactamases are divided into four classes (A, B, C, and D) consisting of two major families: serine-β-lactamases (SBLs) and Metallo-β-lactamases (MBLs)^3,4^.

SBLs (A, C, and D Ambler’s class) are serine hydrolases, while MBLs (B class) perform zinc ion in the active site as metallo-enzyme. Metallo-β-lactamases, owing to their broad-spectrum activities in the hydrolase of β-lactam antibiotics, are a considerable danger for human health.

MBLs are divided into subclasses B1, B2, and B3, and subclass B1 has the most clinical relevance and most commonly emergence among MBLs enzymes. This class has been found in Enterobacteriaceae members, and the enzyme members of this class have two ion zinc in the active site. Some enzymes of subclass B1 consisting of VIM (Verona integron-borne metallo-β-lactamase), IMP (imipenemase), and NDM (New Delhi metallo-β-lactamase-1) have the highest frequency^4^.

First time in 2009, New Delhi metallo-β-lactamase-1 (NDM-1) was identified in *Klebsiella pneumonia* from a clinical sample isolated from a tourist patient in New Delhi^5^. Further assessments showed that this enzyme had high resistance to all classes of beta-lactam antibiotics except monobactams^6^. After the NDM-1 first report, variants of this enzyme emerged in the world and it became a global concern^7^. Furthermore, most plasmids carrying the NDM-1 gene often associate to other resistance genes, such as sulfonamides, rifampin, chloramphenicol, quinolones, and macrolides^8,9^. This property converts NDM-1 carrying bacteria to multi-drug resistant bacteria. Hence, NDM positive strains with these broad resistances to multiple drugs have grown a severe worldwide menace.

The worldwide distribution of NDM-1 has a considerable impact on treating different kinds of infections. Currently, there is no potent inhibitor against this enzyme; therefore, finding an inhibitor for it has become indispensable^10,11^. Natural products (NPs) are a safe resource for human use and are suggested as a valuable source substitute for small molecule drugs. NPs are secondary metabolites derived from natural sources, e.g., micro-organisms, plants and animals, which have valuable and considerable biological activity^12–14^. These molecules have been selected within thousands of years to improve human health. In this study, the Natural Products Activity and Species Source (NPASSv1.0) database is used to screen natural compounds against NDM-1. Currently, the NPASS database consists of 35,032 NPs from different species sources. Traditional chines medicine (TCM) plants and different kinds of NPs and plants are included in this database^15^. Every subclass of MBLs has several types of enzymes and every type has several subtypes of enzymes. There are some FDA-approval small molecules (such as Vaborbactam, clavulanic acid, sulbactam, tazobactam) to inhibit the serine β-lactamases, however these small molecules are ineffective to suppress the metallo-β-lactamases^16–18^.

It seems like common pharmacophore, 3D conformational alignment and fragment molecular orbital calculation are essential to recognize worthy small molecules that are potent to inhibit all types of metallo-β-lactamases. Pharmacophore modeling and 3D conformational alignment across all types of the MBL lead to monitor hot spot or vital residues which are common in the spatial location and sequence position. Finally, FMO and pair interaction energy decomposition analyses were the best option to analyze the crucial conserved residues and confirm the other results. Targeting of these residues by small molecules or natural compounds maybe inhibit all forms of MBLs and terminate domination of them.

Small molecules which must inhibit all sorts of MBLs should bind these common pharmacophores.

The present study aimed to target NDM-1 and all MBLs subclasses and found hotspot residues in all kinds of MBL. These residues can contribute to the better finding of the ligands in order to inhibit all MBLs. Our final screened natural compounds may be effective to suppress MBLs since they had interaction with those residues.

This paper seeks to address the following questions:

1. There is a strategy to block the enzyme's family with a low level of the sequences similarity?
2. Can we discover natural compounds or small molecules that may block all MBLs, like the inhibitors of the SBLs?
3. Can we made a mechanism or algorithm to figure out crucial conserved residues in the enzyme's family to block them?

We believe that we resolved these questions, and may our pathway to investigate crucial conserve residue help other scientists in drug design.

## Methods

**Figure 1:**
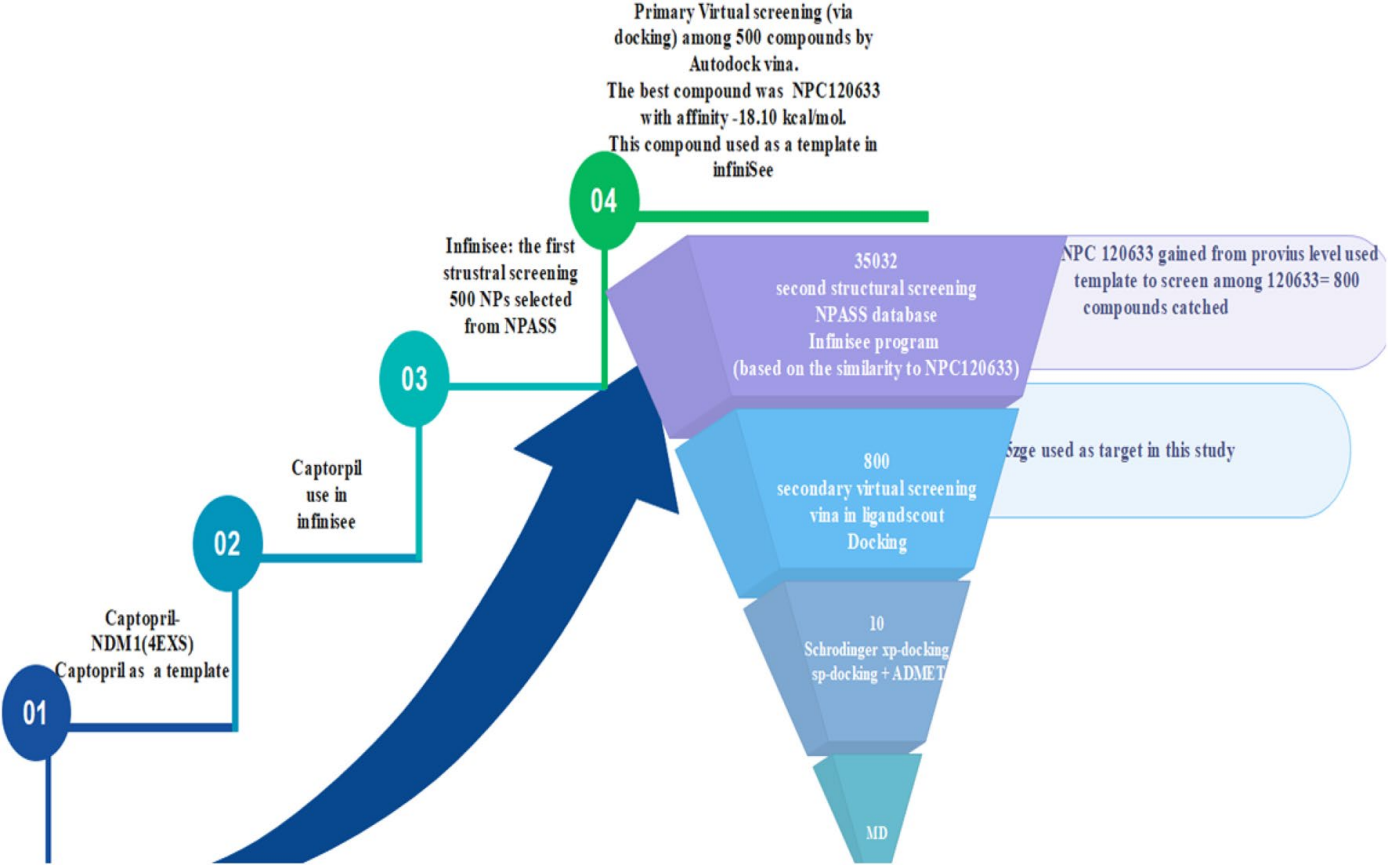
The procedure of this study.

Natural compounds screening steps of this study display in Fig. 1.

### Protein and ligand preparation

High-resolution X-ray diffraction of NDM-1 structures include bound NDM-1 to the hydrolyzed ampicillin (PDB ID: 5ZGE) were downloaded from protein data bank (https://www.rcsb.org/pdb). 5ZGE consist of two NDM-1 molecules bound to the hydrolyzed ampicillin. Among two molecules, chain A was selected, and chain B omitted. NDM-1 enzyme contained 241 amino acids and two zinc ions, initiating from GLU 30 and terminating at ARG 271 position. Captopril was extracted from 4EXS PDB code and use as a template for the first structural screening in the infiniSee 1.3 software.

At first, all water molecules were deleted, hydrogen atoms added with the Discovery Studio software 2.5 (DS, Accerlys Inc, San Diego), and protein prepared for the virtual screening and docking. Energy minimization was done with a simulation module of Discovery Studio via conjugating gradient method and CHARMM force field, until energy gradient fell below 0.1 calÅ^−1^^19^.

### Selection of hit compound and primary virtual screening

In the first structural screening, captopril was used as the template to find hit compound from the NPASS database through infiniSee 1.3 software. This database consisted of 35,032 NPs from different species sources. In this step, 500 compounds were screened among 35,032 compounds. In the next level, these 500 compounds minimized and docked (primary virtual screening) into NDM-1(5ZGE) by Autodock Vina module of the LigandScout.

Among the 500 compounds, NPC120633 had the best affinity of docking via Autodock Vina module of the LigandScout, so NPC120633 selected as the second structural screening pattern in infineSee 1.3 software.

### Second structural screening

Second structural screening was performed to identify hit compounds from the NPASSv1.0 database (http://bidd2.nus.edu.sg/NPASS/) by infiniSee 1.3 software.

This structural screening was done over the NPASS database (similarity 75–100, based on the resemblance to NPC120633 obtained from the previous step) lead to 800 compounds. This collection was minimized by MMFF94 energy method in the LigandScout 4.3 to dock in the Autodock Vina module of the LigandScout^20,21^.

### Molecular docking (secondary virtual screening)

Autodock Vina module of the LigandScout 4.3 was employed to dock all ligands to NDM-1. At first, Autodock Vina was applied for the primary docking of the 800 ligand collection. The applied grid map was at 20 × 20 × 20 points for x, y and z dimensions with grid spacing of 1.0 Å. The center grid box coordinates were placed at 0.772 Å, 51.827 Å, 106.874 Å for the x, y and z-axes, respectively. Default parameters for the Lamarckian genetic algorithm were applied and the numbers of run were on the 40 for profile docking. The profiles of interactions among selected ligands and NDM-1 took through LigPlot and Discovery Studio Visualizer software^19,22^.

Finally, the best compounds which had the proper docking score in the Autodock Vina redocked to NDM-1 by Schrödinger suite software with standard precision (SP) docking and extra precision (XP) docking methods^23^. Redocking process lead to verification of ligands position in active site of protein and redocking process was done to confirm accuracy of visa’s docking score.

All of the selected ligands had very low negative values of the Autodock Vina affinity that have never seen before. It was a significant achievement and beyond expectation, so GRID MIF was performed to pursue this affinity origin. GRID is an enormously well-attested approach to determine thermodynamically advantageous interaction sites on molecules of known structure. Generated molecular interaction fields (MIFs) via GRID force-field define the strength and direction types of molecular interactions in the complex active site. MIF is a three-dimensional (3D) potential interaction energy map among the ligand molecule and probe molecule. This method is frequently employed as a descriptor to form a quantitative structure–activity relationship (QSAR) models^24^.

FLAP software was used to create GRID MIF calculation. The reasonable probes for ligand binding in protein which is adequate to use in MIF consist of the N1 probe (amide nitrogen) to exhibit hydrogen bond donor groups, the O probe (carbonyl oxygen) to describe hydrogen bond acceptor groups, the DRY probe representing hydrophobic interactions, the CRY show Mixed hydrophobic/lipophilic, the C1 to describe aromatic probe. Grid-spacing of 0.5 Å was applied with the pocket point radius of 7 Å beyond a molecule and resolution of 0.5 Angstrom^25^.

### ADME/T properties

The best compound which had proper docking score was selected for ADME/T properties estimation. QIKPROP module of the Schrödinger suite was applied to calculate the ADME/T properties.

### Molecular dynamic simulation

Ten selected NPs which achieved from re-docking and had adequate docking score passed to this step.

The molecular dynamic simulations were used by AMBER18 package, and LEAP module performed to add hydrogen to atoms. AM1-BCC method of the Antechamber module was performed to produce NPs charge. The topology of macromolecules was generated via the leap module of AMBER tools with the AMBER99SB force field^26,27^. The generalized AMBER force field (GAFF2) in the Antechamber module of AMBER18 was applied to build the ligands topology. Then complexes were solvated in a truncated octahedral box of TIP3P water molecules with diameter of 10 Å. The boxes were neutralized by one Na^+^ ion.

Two zinc ions in PDB structure connected to NE2 of His 189, ND1 of His 122, NE2 His 120, NE2 of His 250, OD2 of Asp 124 and SG of Cys 208 via tleap module of Amber18.

For each system, sander module was used for energy minimization. Ten thousand cycles of minimization were done to delete the bad steric interactions and make better minimum energy via the conjugate gradients and steepest descent for all parts of the systems. Thereafter, the position restraints were performed at the constant volume (NVT) for 100 ps by a restraint force of 10 kcal/mol at the temperature of 100 K, and at constant pressure (NPT) for 100 ps by a restraint force of 1 kcal/mol at the temperature of 300 K.

Next, the system was equilibrated at NPT ensemble in density for 100 ps at pressure 1 atm and 300 K, while the restraint force was removed. This method led to equilibrate density for the system. Langevin dynamics were applied to control the temperature of systems.

Fifty nanosecond MD simulation with time step 2 fs in the final step was performed for each protein–ligand complex. CPPTRAJ of AMBER18 was used to analyze MD simulations.

### Binding energy and thermodynamic parameter computation

Molecular Mechanics/Poisson-Boltzmann Surface Area (MM-PBSA) and Molecular Mechanics-Generalized Born Surface Area (MM-GBSA) are effective methods in Computer-Aided Drug Design (CADD) to determine of the binding free energy. The binding free energy for each complex was calculated with MMPBSA.py script in AMBER18 package using below equation.

$$\Delta {\text{G}}_{{{\text{binding}}}} = {\text{G}}_{{{\text{complex}}}} - {\text{G}}_{{{\text{protein}}}} - {\text{G}}_{{{\text{ligand}}}} .$$

The estimation of the binding free energy by MM-PBSA and MM-GBSA in the molecular dynamics simulations showed docking precision. The last ten ns of MD simulation trajectories were used to calculate the binding free energy.

### Vital residues determinate step

**Figure 2:**
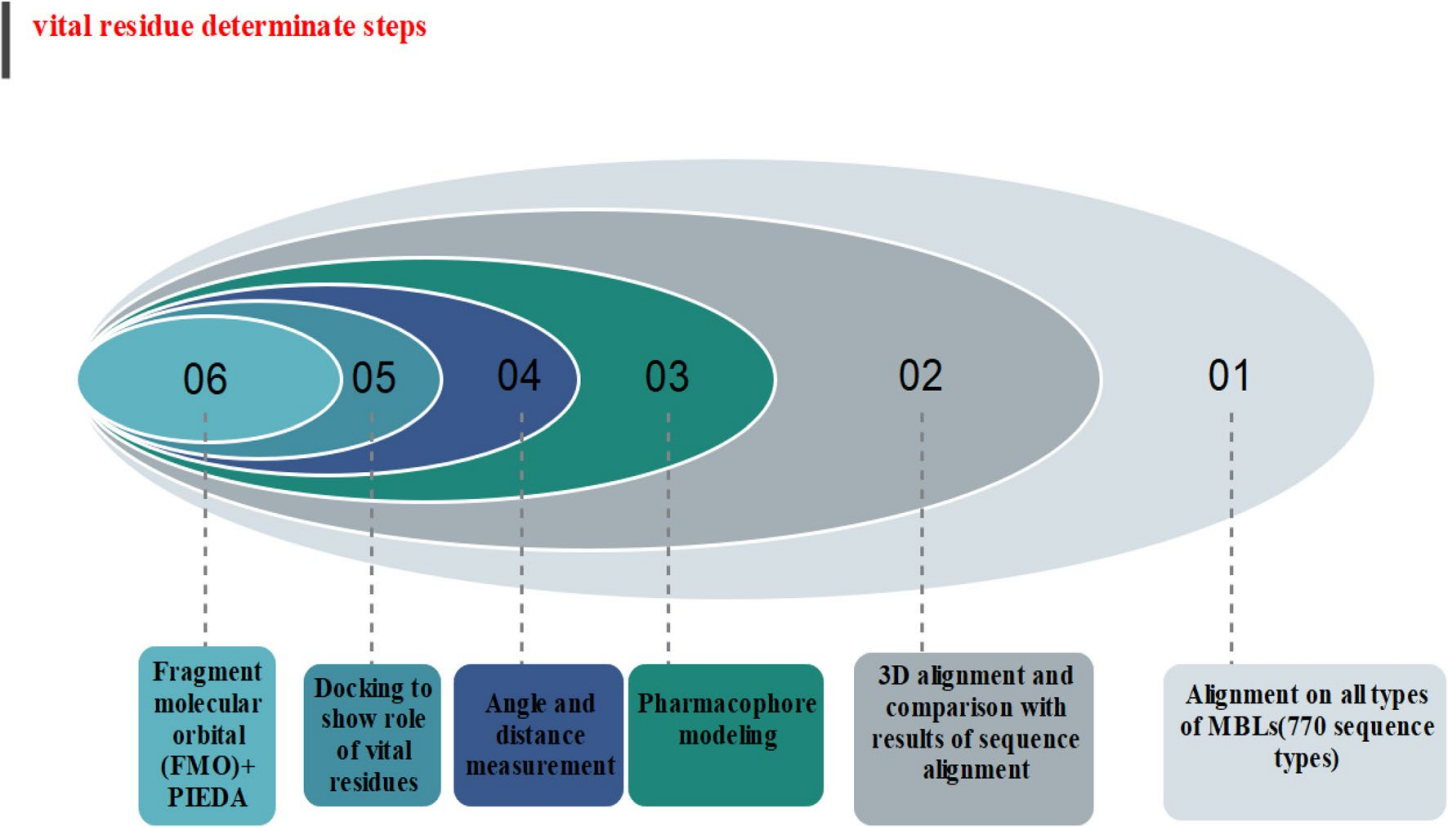
Common pharmacophore and conserve key amino acids in active site of all MBLs subclasses were found via pharmacophore modeling, 3D conformational alignments, protein sequence alignments over MBLs subtypes (more than 700 subtypes) and assessment of distances, angles between crucial amino acids. Comparison of these data showed common pharmacophore and key amino acids among all class of MBL was matchable and compatible. In order to confirm the efficacy of above data, biapenem was docked in the three classes of MBL. Docking and post docking process showed key residues had involved in the docking and all three docking results had common pharmacophore. FMO is the uttermost of assessment to vouch and confirm achievement of other phases.

The flowchart of the discovery process of conserved vital residue in this study is shown in Fig. 2.

### Proteins sequences alignment

Metallo-β-lactamases have three subclasses include B1, B2 and B3. Every subclass has several types of enzymes and every type has several subtypes of enzymes. MBLs comprise more than 770 subtypes (B1 = 507, B2 = 22, B3 = 248).

Separate alignment among every subclass was performed by BLDB databases (Beta-Lactamase DataBase—Structure and Function; http://bldb.eu/), and conserve residues were selected, then alignment across three subclasses showed the conserved residues among them.

### 3D conformational alignment

This sort of measurement aims to discover some residues that may not conserve in the protein sequence alignment but are analogous to two types of enzymes.

3D conformational alignment of the structures of representative kinds of MBLs (B1 = NDM1–B2 = BJP–B3 = Cph) was done by the chimera software to investigate spatial orientation and sequence location of conserved residues which obtained from proteins sequence Alignment level. Simultaneously conserved residues with an equivalent spatial orientation and spatial location in three subclasses' active sites were chosen and screened to pass the next level.

### Pharmacophore modeling

Distances and angels among the amino acids of active sites manually computed in the LigandScout software and pharmacophore modeling were done between representative kinds of MBLs. These data were compared with each other.

### Docking with biapenem

Three typical patterns of MBL classes (NDM1-BJP-Cph) docked with biapenem in autodock vina and post-docking processing done by LigPlot. This stage is a complement to the other steps.

### FMO and PIEDA

To more in-depth calculation, quantum mechanics computation with fragment molecular orbital (FMO) method was performed to evaluate pair interaction energy decomposition analyses (PIEDA) among the ligand and protein in downloaded complex.

FMO method was described by Kato et al. and Sliwa et al.^28,29^. In summary, on the NDM and CPH and BJP proteins, protonate 3D, N- and C-termini capping and energy minimization with AMBER10: EHT force field was done via MOE program.

The water and ion molecules were deleted. Input file created by Facio program through automatic method fragmentation^30^. FMO calculation was RHF/6-31G* in GAMESS program^31^. Pair interaction energy decomposition analysis was analyzed in Facio program. Four energy components that were assessed contain electrostatic (ES), exchange repulsion (EX), charge transfer with mixed term (CT mix) and total pair interaction (Etot) contributions.

Two different ligands were selected to comprehensively compare and more profoundly vision the interaction among MBLs and their ligands. If the vital residues in various MBLs types had significant PIEDA participation with divers beta-lactam, these results vigorously verify prior levels' outcomes.

## Results

### The virtual screening

**Figure 3:**
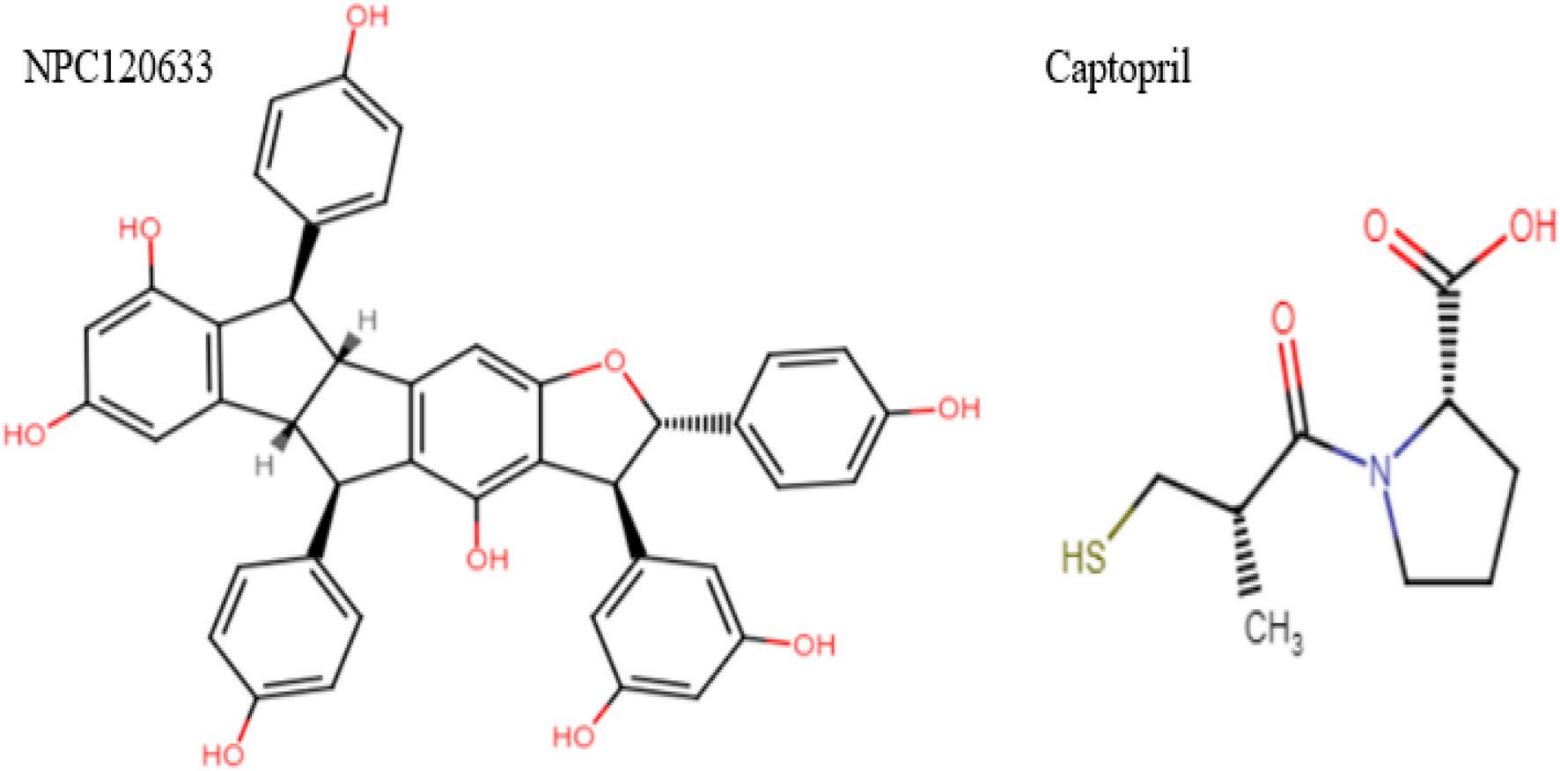
The structure of captopril and NPC120633.

In the first structural screening, captopril is performed as the template to find the hit compound from the NPASS database via infiniSee 1.3 software. In this step, 500 compounds were screened. These compounds were minimized and docked to NDM-1 by Autodock Vina module of LigandScout 4.3. Among the 500 compounds, NPC120633 had the best binding affinity energy of − 18.1 kcal/mol, so NPC120633 selected as the second structural screening pattern in the infineSee 1.3 software. The structure of captopril and NPC120633 were shown in Fig. 3.

### The docking result

**Figure 4:**
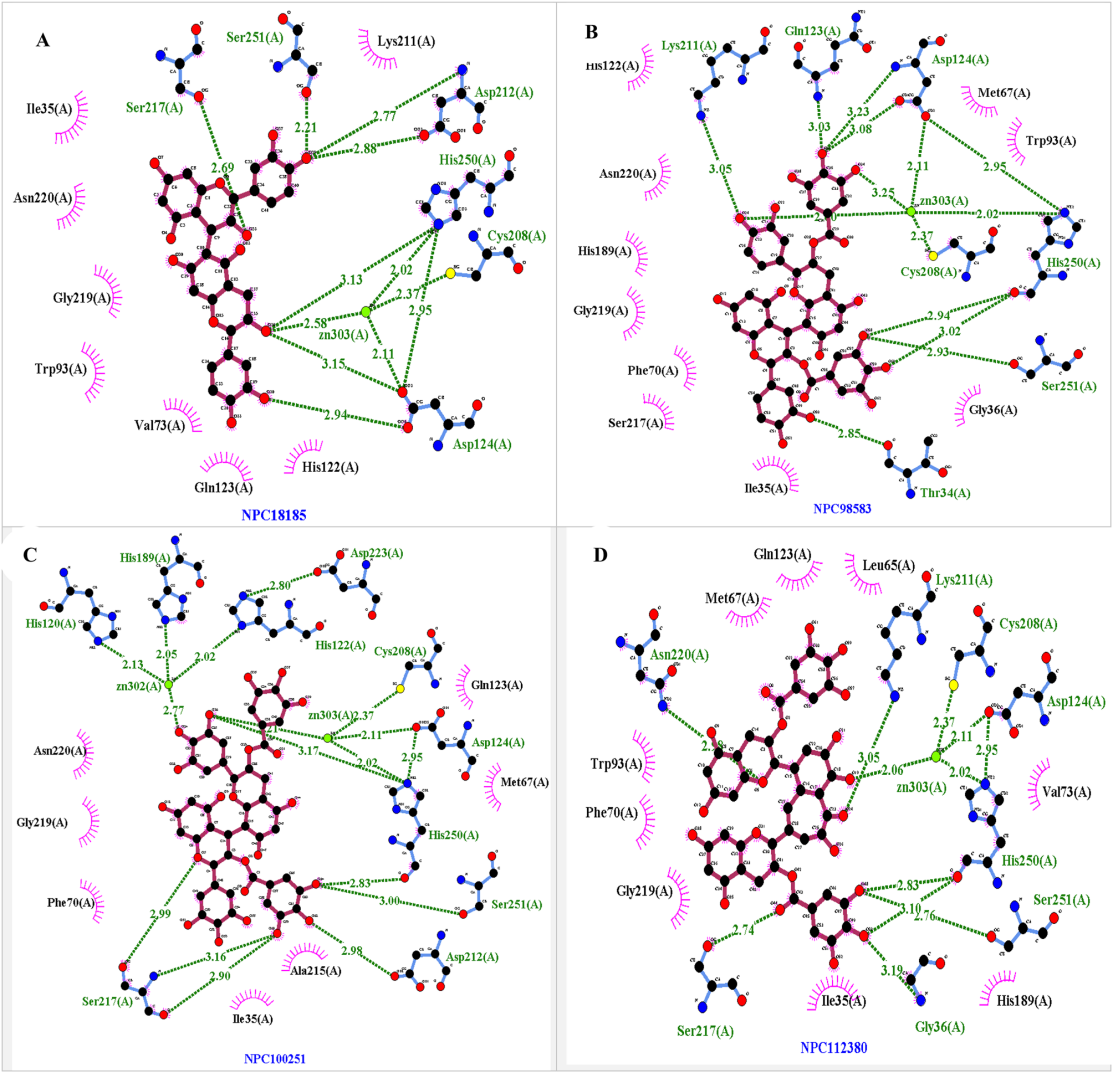

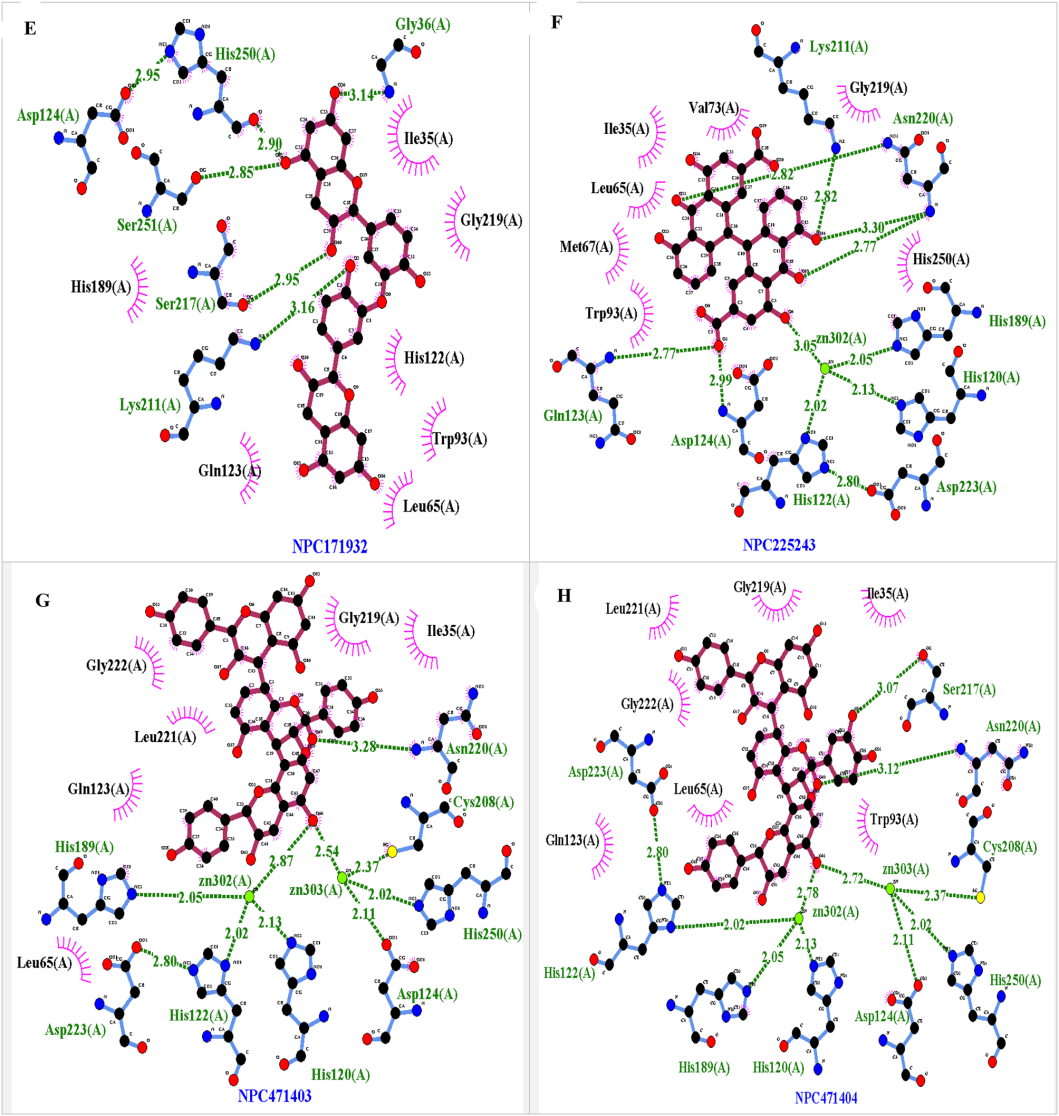

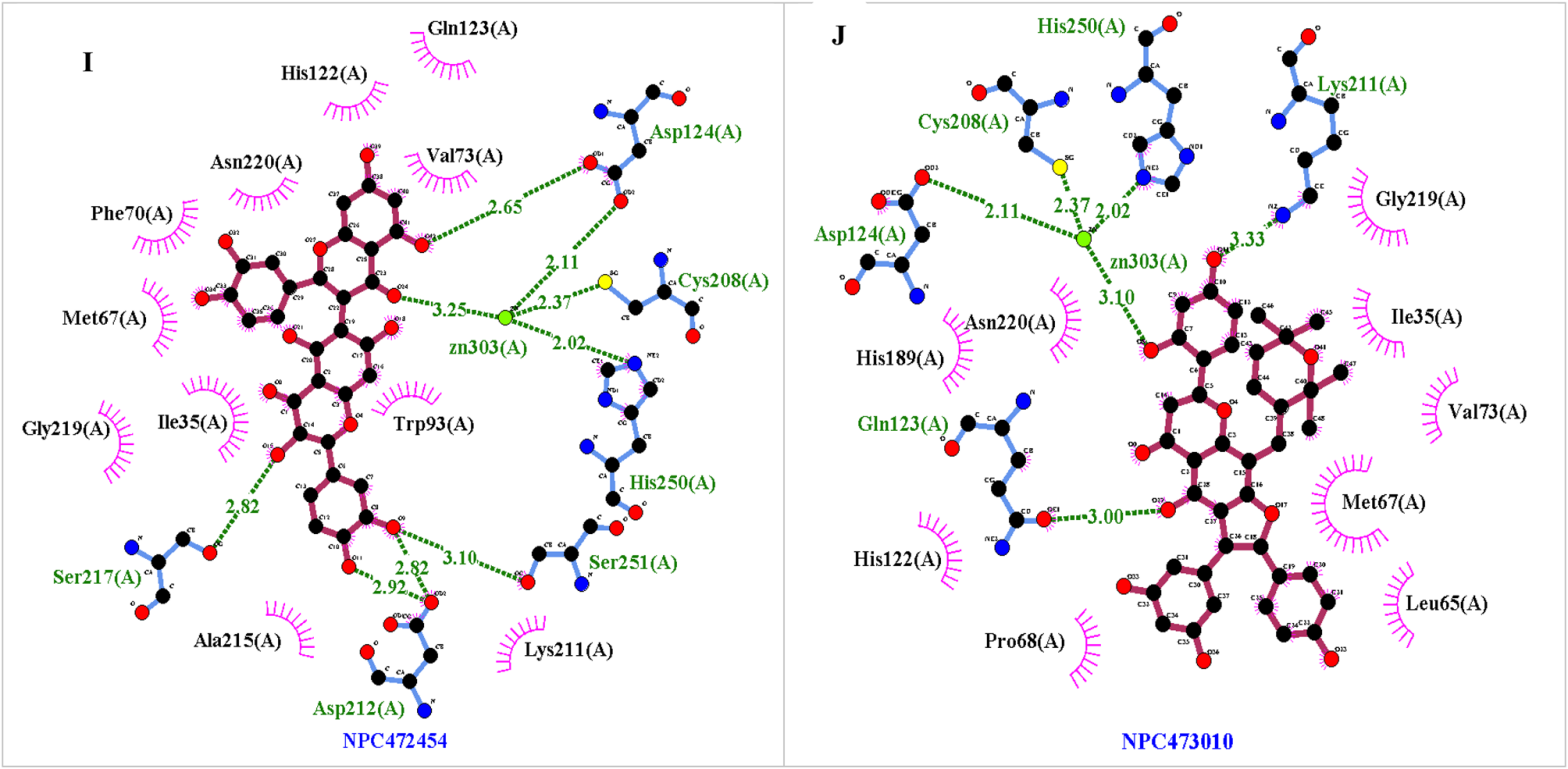
The predicted complex NDM1-NPC binding mode. The position of the NPs in the active site of the NDM, hydrogen bonds and hydrophobic interactions were shown. (**A**) NDM1-NPC18185, (**B**) NDM1-NPC98583, (**C**) NDM1-NPC100251, (**D**) NDM1-NPC112380, (**E**) NDM1-NPC171932, (**F**) NDM1-NPC300657, (**G**) NDM1-NPC471403, (**H**) NDM1-NPC471404, (**I**) NDM1-NPC472454, (**J**) NDM1-NPC473010.

Eight hundred compounds were docked in second screening step via Autodock Vina and among them, ten compounds had the proper and higher affinity binding. The binding energy of ten selected NP obtained from Vina software, i.e. NPC171932, NPC100251, NPC18185, NPC98583, NPC112380, NPC471403, NPC471404, NPC472454, NPC473010 and NPC300657 were − 24.3 kcal/mol, − 22.9 kcal/mol, − 22.8 kcal/mol, − 22.2 kcal/mol, − 22.80 kcal/mol, − 21.8 kcal/mol, − 22.3 kcal/mol, − 22.7 kcal/mol, − 21.3 kcal/mol and − 20.8 kcal/mol respectively. In the Schrödinger suite, these compounds had adequate sp- and xp-docking score (Table 1). The corresponding docking binding affinity $k_{b}$ of all ligands were at the high level (Table 1). Binding affinity energy of Vina and XP and SP Glide score and hydrogen bonds and hydrophobic contacts were exhibited for all ligands with protein were mentioned in Fig. 4 and Table 1. Hydrogen bonding and hydrophobic interactions of the complexes and distance between atoms ligands and protein were shown in Table 2.

**Table 1:** The binding energy of Autodock Vina and Schrodinger suite of all NPs. Glide uses Emodel to pick the "best" pose of a ligand (pose selection), and then ranks these best poses against one another with GlideScore. *TC* Tanimoto coefficient score.

Complex	Structure	Glide XP-Score (kcal/mo)l	Glide E-Model	Glide SP-Score (kcal/mol)	autodock vina (kcal/mol)	TC	K_b_ (M^−1^)
NDM1-NPC18185		− 10.00	− 64.58	− 7.51	− 22.8	0.23	4.6 × 10^16^
NDM1-NPC98583		− 7.78	− 62.64	− 7.15	− 22.2	0.27	1.7 × 10^16^
NDM1-NPC100251		− 13.95	− 100.1	− 8.32	− 22	0.22	1.2 × 10^16^
NDM1-NPC112380		− 12.76	− 89.03	− 8.59	− 22.80	0.28	4.6 × 10^16^
NDM1-NPC171932		− 8.395	− 86.56	− 8.12	− 24.3	0.22	5.8 × 10^17^
NDM1-NPC471403		− 9.036	− 71.40	− 7.07	− 21.8	0.20	9.6 × 10^15^
NDM1-NPC471404		− 8.561	− 72.09	− 8.53	− 22.3	0.20	2 × 10^16^
NDM1-NPC472454		− 10.06	− 46.60	− 7.37	− 22.7	0.25	3.9 × 10^16^
NDM1-NPC473010		− 7.01	− 62.85	− 7.11	− 21.3	0.30	3.7 × 10^15^
NDM1-NPC300657		− 10.068	− 91.49	− 8.10	− 20.8	0.25	1.6 × 10^15^

**Table 2:** Hydrogen bonding and hydrophobic interactions of the complexes and distance between atoms ligands and protein.

Complex	Hydrogen bonds		Zinc interactions		Hydrophobic interaction	
	Residue	Distance (Å)	Zincs	Distance (Å)	Amino acids	Distance (Å)
NDM1-NPC18185	Asp212, Ser217, Ser251, His250, Asp124	2.21–3.13	Zinc303	2.58–3.15	His250, Ile35, Gly219, Asn220 ,Val73, Trp93, Gln23, His122, Asp124, Ser251, Lys211, Asp212, Ser217	3.30–4.30
NDM1-NPC98583	Gln23, Asp124, His250, Ser251, Thr134, Lys211	2.60–3.25	Zinc303	2.60–3.25	Ile35, Lys211, Gly36, Phe70, Ser217, Gly219, Lys211, His189, Asn220, Trp93, Met67, Gln23, Asp124, His122, His250, Ser251	3.42–4.02
NDM1-NPC100251	Ser217, Asp212, Ser251, His250	2.13–3.16	Zinc302–Zinc303	2.21–2.77	Ser217, Gly219, Asn220, Phe70, His189, Gln123, Met67, Ile35, Ala215	3.4–4.40
NDM1-NPC112380	Ser217, Gly36, Ser251, His250, Lys211, Asn220	2.06–3.19	Zinc303	2.06	Asn220, Val73, Trp93, Gln23, Lue65, His189, Asp124, His250, Cys208, Lys211, Gly36, Ser251, Ser217, Ile35, Gly219	3.46–3.9
NDM1-NPC171932	His250, Ser251, Gly36, Lys211, Ser217	2.85–3.16			His250, Gly36, Ile35, Trp93, Lue65, Gln123, His122, Lys211, His189, Ser217, Gly219	2.88–4.4
NDM1-NPC471403	Asn220	3.28	Zinc302–Zinc303	2.54–2.87	Asn220, Cys208, Asp124, His189, Gln123, Lue65, Gly222, Lue221, Gly219, Ile35	3.8–5.6
NDM1-NPC471404	Asn220, Ser217	3.07–3.12	Zinc302–Zinc303	2.72–2.78	Asn220, Ser217, Ile35, Trp93, Asp124, His189, Lue65, Gln123, Gly222, Lue221, Gly219	3–4.69
NDM1-NPC472454	Asp124, Ser251, Asp212, Ser217	2.65–3.25	Zinc303	3.25	Ile35, Ser251, Lys211, Asp212, Ala215, Gly219, Phe70, Met67, Asn220 Gln123, His122, Trp93, Asp124	3.74–5.1
NDM1-NPC473010	Lys211, Gln123,	3–3.33	Zinc303	3.10	His250, Lys211, Gly219, Ile35, Val73, Met67, Lue65, Pro68, Gln123, His122, His189, Asp124, Asn220	3.36–4.69
NDM1-NPC300657	Asp124, Gln123, Lys211, Ser217	2.82–3.19	Zinc303	2.83	Asp124, Trp93, His250, Lue221, His189, Lys211, Lue221, Ser217, Phe70, Met67, Ile35, His122, Gln123	3.1–3.9

Figure 4 shows the predicted complex NDM1-NPCs binding mode and the position of the NPs in the active site of the NDM1, hydrogen bonds and hydrophobic interaction of NPs with active site residues of NDM1 were shown. This Figure was obtained via LigPlot software and confirmed above data about the crucial residues' key role in all complex. Crucial residues existed and involved in the ligand binding interactions.

Hierarchical clustering of compounds in Figure S1 (supplementary) shows that final compounds had good diversity. Tanimoto coefficient score of ten selected molecules were in the range of 0.2–0.3 in comparison to NPC120633 as references molecules (as TC in Table 1).

five and six O atoms of NPC171932 ligand had overlap to O and N probe in MIF respectively, however, it had five hydrogen bonds. Notice that the NPC100251 six and seven O atoms of NPC171932 matched well to the O and N probes in the MIF, respectively, although it made five interactions with the protein in these positions. The DRY probes that characterizes the hydrophobic interactions overlapped well with two or three aromatic rings in all ligands. The CRY probes showed very similar interactions to that of the DRY.

**Figure 5:**
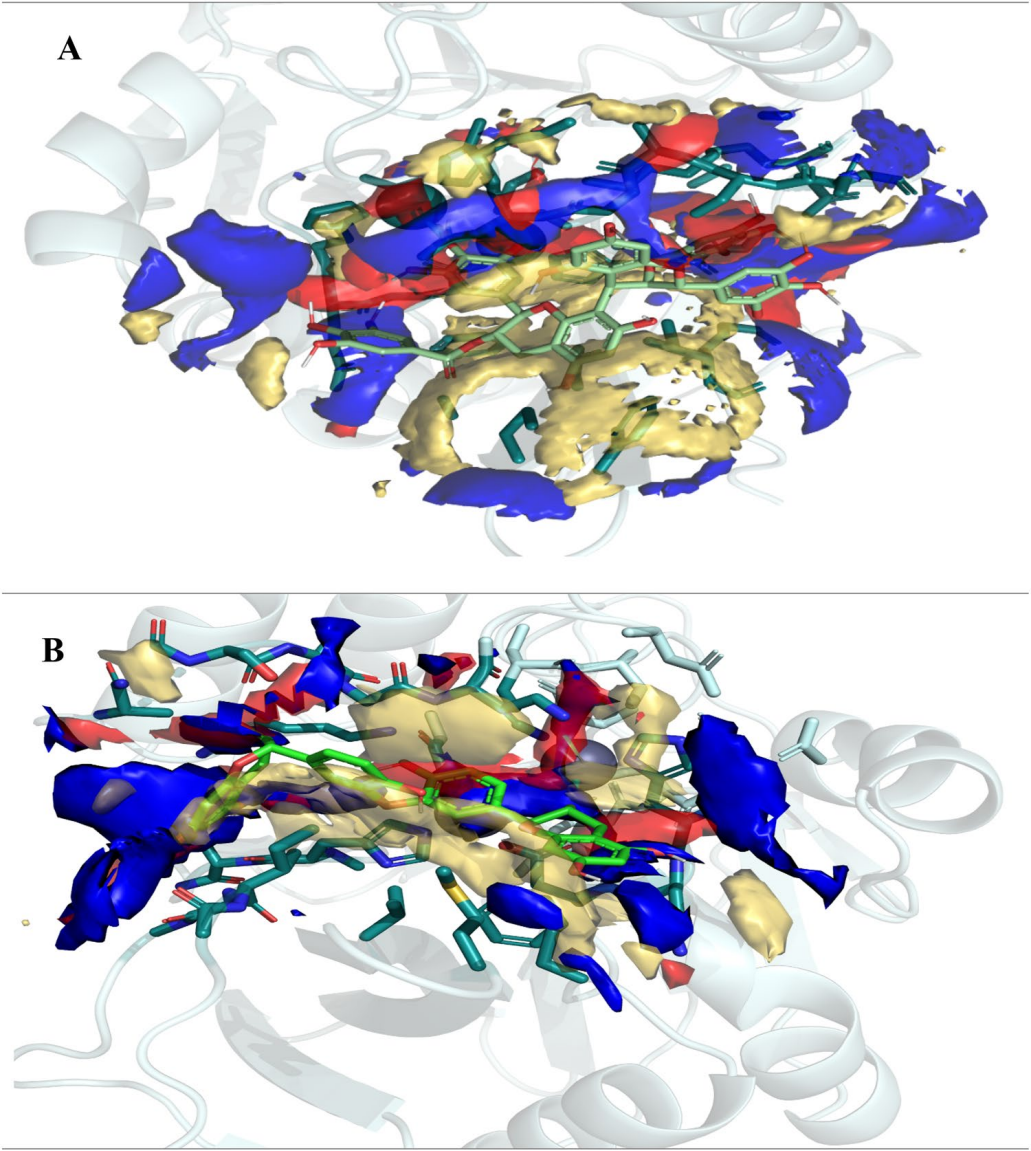
A-GRID MIFs of complex NDM-NPC100251 B- GRID MIFs of complex NDM- NPC171932. GRID MIFs were used for probes O (red, − 3.5 kcal), N1 (blue, − 3.5 kcal), DRY (brown, − 0.5 kcal). Values of 0.0 kcal/mol to < − 2.5 kcal/mol tend to show the non-polar interactions, values of > − 2.5 kcal/mol tend to indicate hydrogen bonding is occurring, a and more negative values start to illustrate stronger charge interactions. Most ligand functional groups are associated in the O or N GRID MIF with 3.5 kcal/mol in the picture. Aromatic rings engaged in the DRY and CRY with 0.5 kcal/mol MIF. Some of oxygen in the ligand hydroxyl group had electrostatic contacts with Zinc ions. ΔΔ G binding = ΔΔG hydrogen bond + ΔΔG hydrophobic + ΔΔG vdw + ΔΔG electrostatic. It seems like the high number of the hydrogen bond, very low-value energy of the hydrophobic bond, van der Waals (near to hydrogen bond energy level) made those docking scores.

In all ligands, O atoms had several contacts with O and N probes MIF. These contacts were two-fold more than of the hydrogen bonds. It stated those overlapped O atoms made some hydrophobic interaction with a negative value near of hydrogen bond. Summation of all hydrogen bonds, hydrophobic and lipophilic interaction produced such a low-affinity range (Fig. 5).

### Physio-chemical and ADME/T properties

Physio-chemical and the ADME/T properties of the ten docked NPs were calculated via ligandscout and QIKPROP of Schrödinger suite. Physio-chemical were indicated in the Table S1. ADME/T properties consisted of the values for polarizability (QPpolrz), aqueous solubility (QPlogS), hexadecane/gas (QPlogPC16), water/gas (QPlogPw), octanol/water (QPlogPo/w), skin permeability (QPlogKp), and Khsa serum protein binding (QPlogKhsa) which have standard ranges as 13 to 70, − 6 to 0.5, 4 to 18, 8 to 43, − 2 to 6, − 8.0 to − 1.0, and 1.5 to 1.2, respectively^32^. The QIKPROP analysis exhibited values of QPpolrz, QPlogS, QPlogPC16, QPlogPoct, QPlogPw, QPlogPo/w, QPlogKp, and QPlog Khsa were in the standard range (Table 3).

**Table 3:** ADME/T properties: adsorption, distribution, metabolism, excretion, and toxicity.

Entry name	QPpolrz	QPlogS	QPlogPC16	QPlogPw	QPlogPo/w	QPlogKp	QPlogKhsa
NPC18185	44.271	− 2.1	16.269	36.295	− 2.452	− 7.33	− 0.624
NPC98583	65.081	− 0.639	24.495	57.852	− 5.536	− 9.789	− 1.811
NPC100251	65.355	− 0.174	25.598	64.181	− 6.833	− 11.062	− 1.835
NPC112380	61.805	− 0.637	24.609	58.396	− 6.621	− 11.269	− 2.044
NPC171932	43.451	− 1.93	15.271	31.285	− 1.519	− 6.286	− 0.596
NPC300657	63.983	− 4.051	20.104	35.85	− 0.646	− 8.053	− 0.351
NPC471403	67.624	− 3.705	20.691	38.097	− 0.23	− 7.17	− 0.268
NPC471404	65.069	− 2.684	20.534	41.03	− 1.062	− 7.586	− 0.306
NPC472454	45.442	− 2.048	17.118	39.549	− 3.12	− 8.014	− 0.728
NPC473010	62.142	− 4.62	18.407	27.791	1.687	− 5.949	0.193

Based on these analyses, all ten compounds' ADME/T properties have the standard limits to become potential candidate drug compounds.

### Molecular dynamic (MD) simulation

MD simulation is a computational assessment for the stability and dynamics of the ligand-receptor complex under physiological conditions. In this study, MD simulation was performed to access the stability of all ligand-NDM-1 docked complex and their docking poses. Fifty ns of molecular dynamics simulations were applied for all complexes. Backbone RMSD and RMSF of each complex’s trajectory with respect to their initial conformation showed the stability of the complex. The average and standard deviation of backbone RMSD during the last ten ns were shown in Table 4. The small standard deviations indicated that all protein structures reach to stable structures during the last 10 ns of MD simulation.

**Table 4:** The average of backbone RMSD of proteins during the last 10 ns of MD simulation (only the last three digit of name of compounds were mentioned).

	PRO	NPC185	NPC 251	NPC 380	NPC 403	NPC 404
Back-bone RMSD (nm)	0.153 ± 0.015	0.152 ± 0.01	0.141 ± 0.007	0.122 ± 0.027	0.133 ± 0.012	0.115 ± 0.006

	NPC 454	NPC 583	NPC 657	NPC 932	NPC 3010	
Back-bone RMSD (nm)	0.148 ± 0.009	0.122 ± 0.009	0.142 ± 0.011	0.139 ± 0.007	0.167 ± 0.015	

**Figure 6:**
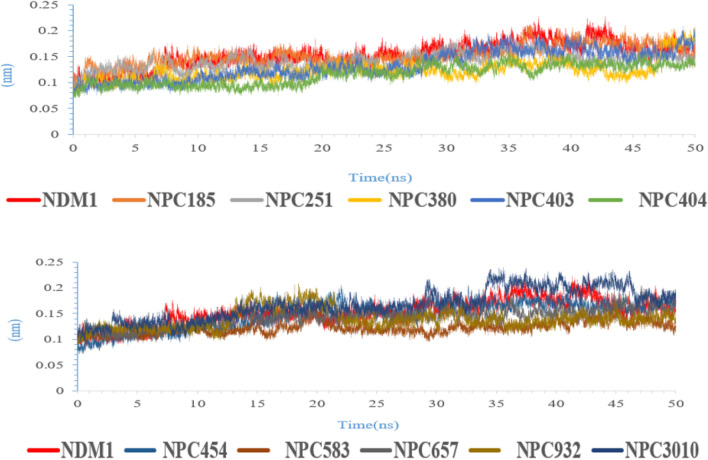
RMSD analyses of ten natural compounds in complex with NMD-1 (only the last three digit of name of compounds were mentioned).

Therefore, it can be inferred NPs could make a stable complex with NDM-1 and inhibit NDM-1 protein. Figure 6 displays the backbone RMSD protein in existence of NPs during 50 ns MD simulation.

**Figure 7:**
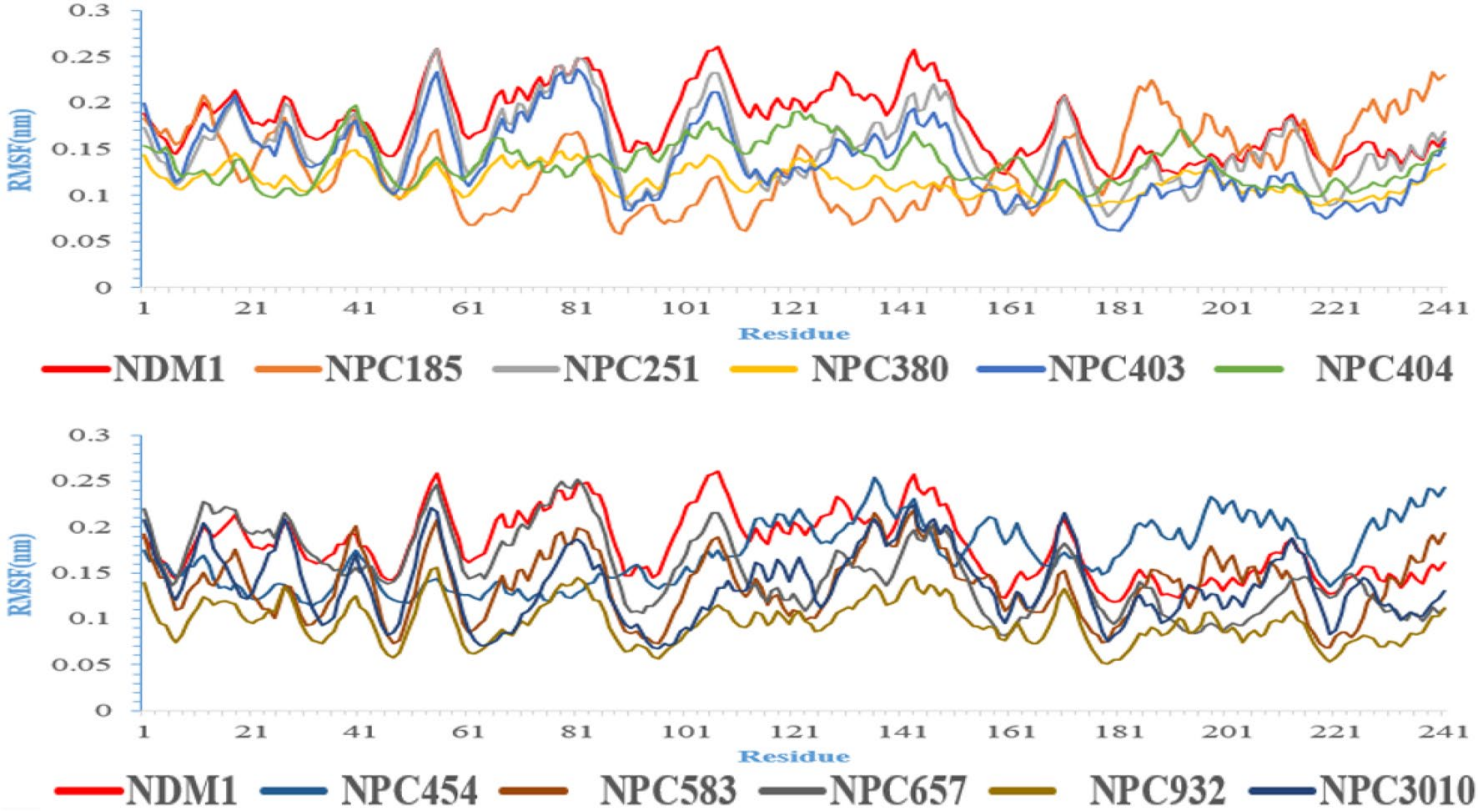
RMSF analyses of all complexes during the last 10 ns of MD simulation.

Figure 7 shows the backbone RMSF plot of proteins in existence of NP compounds. The NDM-1’s residues have lower fluctuation in complex with NPs in comparison to the NDM-1 without ligand (especially amino acids which were involved in the active site of the NDM-1), so these compounds made a stable and robust complex with NDM-1 for inhibition of this enzyme. These results are compatible with the backbone RMSD results.

Table 5 exhibits the value of the MM-PBSA and MM-GBSA binding free energy of every ligand to its protein and also thermodynamics parameters of all complexes. All NPs showed a high binding affinity for NDM-1. Among the compounds, NPC472454 had the lowest binding energy (− 137.12 kcal/mol). Thermodynamic parameters with a negative value of the ΔH and ΔG exhibited the event possibility of the binding between proteins and ligands was exothermic. These results confirm RMSD and RMSF results.

**Table 5:** Thermodynamic parameters of binding ligands to NDM.

Complex	ΔG_MM-PBSA_ (kcal/mol)	ΔG_MM-GBSA_ (kcal/mol)	ΔS (kcal/mol)	ΔH (kcal/mol)
NDM1-NPC18185	− 49.82	− 48.23	− 77.11	− 23,037.18
NDM1-NPC98583	− 49.99	− 53.92	− 110.85	− 33,079.04
NDM1-NPC100251	− 54.45	− 57.43	− 78.81	− 23,551.65
NDM1-NPC112380	− 53.76	− 49.24	− 99.56	− 29,737.57
NDM1-NPC171932	− 55.54	− 58.12	− 79.60	− 23,788.28
NDM1-NPC471403	− 32.07	− 33.80	− 96.80	− 28,892.92
NDM1-NPC471404	− 43.11	− 39.01	− 94.86	− 28,135.89‬
NDM1-NPC472454	− 137.12	− 136.83	− 74.40	− 22,319.48‬
NDM1-NPC473010	− 75.84	− 74.40	− 92.56	− 27,672.60
NDM1-NPC300657	− 56.75	− 54.99	− 87.53	− 26,213.44

## Vital residue determinate step results

### Proteins sequences alignment

Alignment among sequences of MBLs illustrated some conserved sequences which were in the active site. These sequences and their analog are in the Table 6 and Figure S3. These amino acids pass to the next levels.

**Table 6:** Crucial amino acids and their analogous amino acids in different types of MBLs.

	Crucial amino acids									
B1 = NDM-1	Ser217	Ser251	Asn220	Asp124	His250	Lys211	Ile35	His189	Gly219	His122
B2 = cphA		SER265	Asn233	Asp120	His263	Lys224		His196	Gly232	His118
B3 = BJP-1				Asp105	His242			His177		His103

### 3D conformational alignment, docking with biapenem, pharmacophore modeling and distance and angle measurement

**Figure 8:**
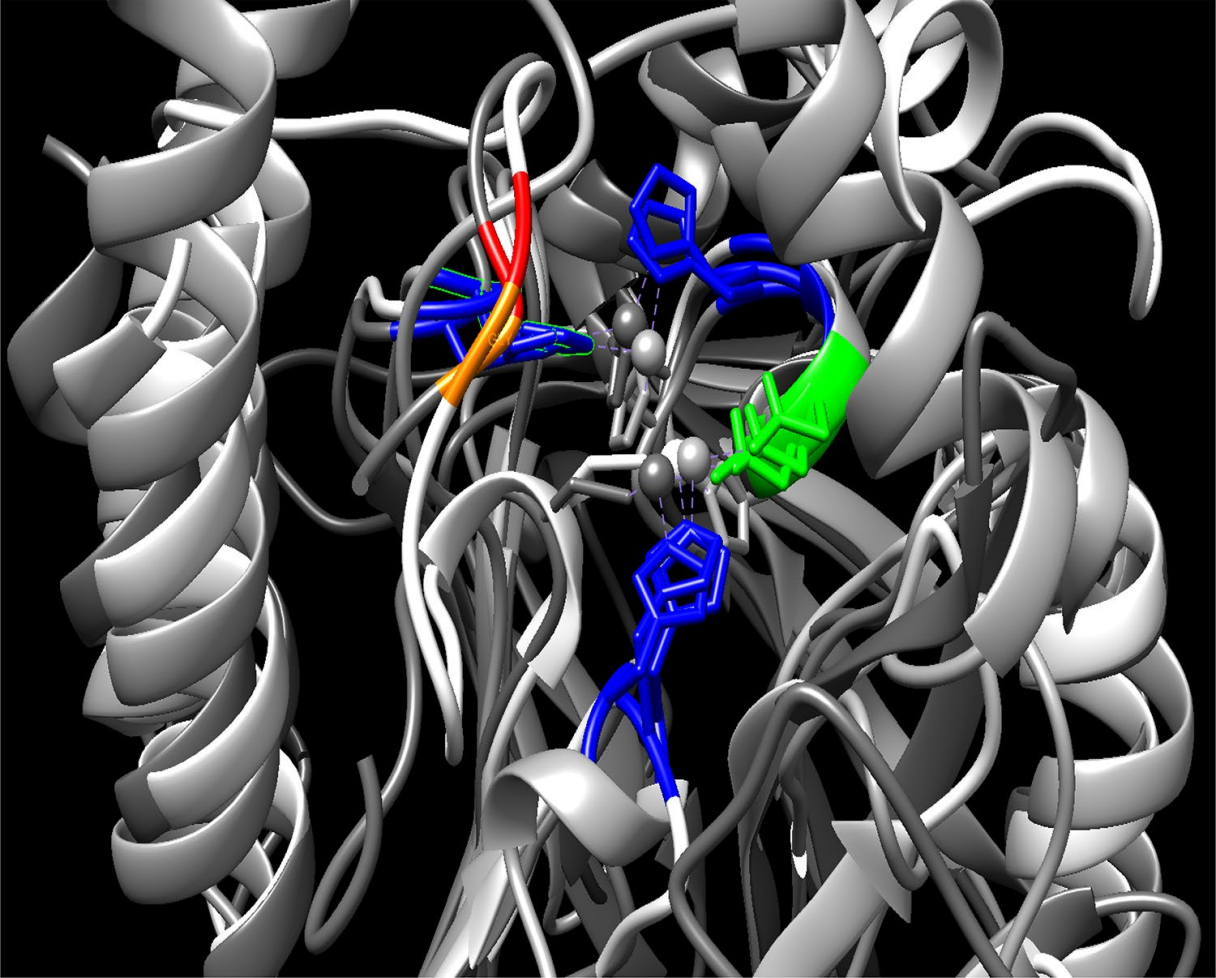
Crucial residue in active site, which conserve in the MBL. These amino acids pass from protein sequence alignment level. Histidine, Aspartic acids, Gly and Asn residues are colored Navy blue, green, orange and red, respectively.

3D conformational alignment demonstrates amino acids in the active sites which were analog in the three subclasses (Figure S2). Figure 8 displays these conserved amino acids. This process exhibited that analogous residues which obtained in protein sequence alignment are very approach.

Docking process was applied on the NDM (PDB = 5ZGE) and BJP-1 (PDB = 5WCM) with biapenem. For cphA (PDB = 1X8I), docking process is not necessary because it had biapenem in pdb complex. Interaction among biapenem and complexes is in Figure S4, S5 and S6. A comparison of these interactions displayed the importance of the aforesaid amino acids in hydrogen bonds and van der walls interaction. These contacts and bonds had real conformity to interaction with ampicillin-NDM, which concluded that interactions MBLs with different beta-lactam antibiotics were the same. On the other hand, all MBLs involve their described residues in the hydrolyze of different ligands.

Distance and angle assessment among equivalent mentioned residues displayed similar and comparable value. Distance and angle present in Figure S7 and S8.

Pharmacophore modeling of three subclasses and synchronization of results revealed functional groups and their pharmacophores had overlap (Figure S9).

Crucial conserved residues in all metallo-β-lactamase classes were identified (Table S2). Comparisons of 3D conformational alignment, protein sequence alignment over 700 subtype of MBL, pharmacophore modeling, distance and angles measurement displayed that crucial residues of metalo-β-lactamase classes were the same and similar (Figure S8, S9).

### FMO and PIEDA

**Figure 9:**
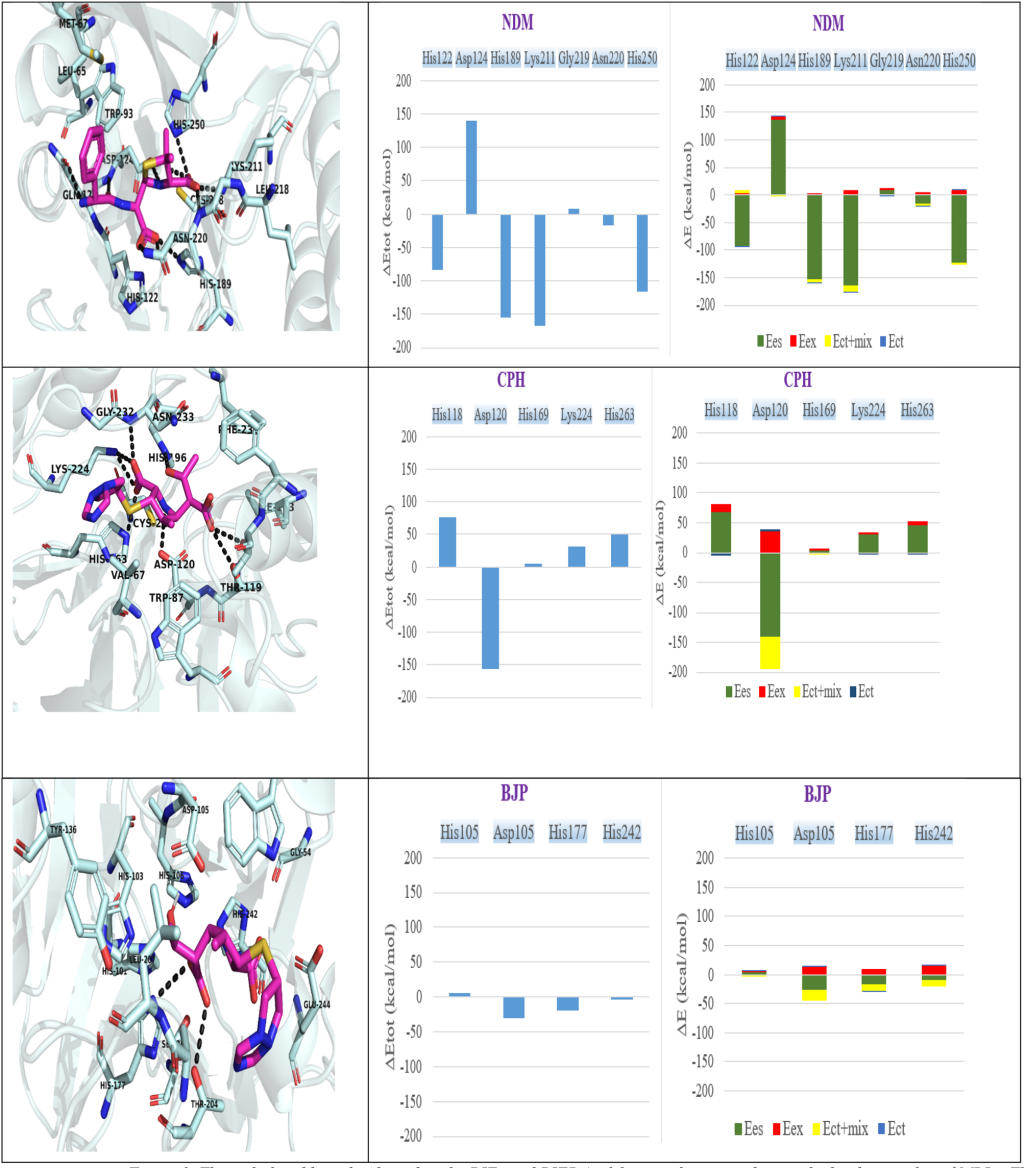
The right hand bar plot describes the PIEs and PIEDA of the significant residues in the binding pocket of MBLs. The electrostatic, exchange repulsion, charge transfer, Ect + mix and E total terms are coloured in green, red, dark blue, yellow, light blue, respectively.

PIEDA finding was mentioned in the Fig. 9. Residues involved in the interaction with ligands approved outcomes of the previous stage. For NDM (complexed with hydrolyzed ampicillin in pdb = 5zge), His122, Asp 124, His189, Lys211, Gly219, Asn220 and His 250 had a more significant contribution than the other residues in the interaction with ligand. amino acids of CPH consist of Asp120, His263, Lys224, His196, Gly232 and His118 residues had a high-level interaction to biapenem. Asp105, His242, His177, His103 in BJP were involved in the interaction with biapenem. The contribution of the critical residues in PIEDA was more considerable than the other residues in three subclasses. All three computations of the FMO clearly revealed that conserved vital residues had a major ES and non-negligible CT contributions, indicating strong attractive interactions hydrogen bonds among these residues and beta-lactam antibiotics.

## Discussion

Global contamination of NDM-1 has become a major threat to human health. Currently, there is no NDM-1 clinically inhibitor. The discovery and development of an inhibitor for inhibiting of NDM-1, as well as restituting and reinforcing the efficacy of the beta-lactam antibiotic against this enzyme would have a significant effect on human health and create obvious socio-economic benefits^17^. These limitations and uttermost necessity inspired us to mine a new lead compound against NDM-1 from natural resources^33^. The reconnaissance of novel non-covalent inhibitors of β-lactamases is an optimistic path to preserve and restore the efficacy of β-lactam antibiotics.

Against this study, different and frequent studies have investigated some chelator compounds of the zinc of NDM and MB, but some studies indicated that mutation in non-active site residues sequences over some MBLs could severely decrease the dependency of these enzymes on zinc ions, therefore, concentrating on the metal’s chelators are not proper option. Even in the starvation of zinc and utilization of EDTA as a metal chelator, MBLs was powerful enough to degrade beta-lactam rings^34–36^.

Other studies examined the compounds affecting one type of the MBL subclasses alone. During those studies, no effort was made to find common pharmacophore and crucial residues playing a key role in the active site (residues conserved in the active site of all kinds of MBL) in all MBL classes^17^. In this study, regarding the above-mentioned weakness, the crucial conserved residues (hotspots residues) participate a significant role in the active site and common pharmacophore in all MBLs were detected.

The NPASS database was used as the database, which had a complete collection of 35,032 natural compounds. For this purpose, 35,032 compounds were screened, and 1300 compounds were docked. Two structural screening steps were done to found the best ligands with a high ability to inhibit the NDM1. At first, captopril was used in structural screening and NPC120633 ligand was obtained. To catch a better ligand with lower binding free energy, second screening based on the similarity to NPC120633 was performed and ten ligands with lower binding energy than NPC120633 were found.

Multi-step molecular docking assisted to facilitate the virtual screening process by yielding insight into the ligand-binding energy and docking binding affinity. Multiple molecular docking and structural screening processes were applied via Autodock Vina (module of LigandScout) and infiniSee software, respectively, and helped to identify the best NPs that could perfectly inhibit NDM-1. Schrödinger suite program was applied to confirm secondary virtual screening (secondary docking) results. Schrödinger results approved the Autodock Vina results for ten final compounds. Validation of docking was confirmed by using Glide XP-docking and Glide SP-docking.

According to the GRID MIF assessment in every ligand, several very low negative value interactions that had overlap with the MIF probe of the NDM-1 existed. The number of these interactions were more than hydrogen bonds in every ligand. For example, in NPC171932 logand, despite five hydrogen bonds, some interactions did not involve the hydrogen bonds and had energy quality near hydrogen bond, so these parts reinforced the affinity among ligand and NDM-1. The MIF calculation illustrated that in most ligand most polar groups participated in contact, Lipophilic groups placed in a lipophilic space of active site, Optimal steric fitted between ligand and receptor occur, and clashing interaction were not generated.

These findings of the current study are consistent with those of kuntz et al. and Carlson et al. who found binding free energy will decrease with the number of the non-hydrogen atoms and the functional group number per atom. These natural compounds with many aromatic rings and hydroxyl functional groups made great affinity with the NDM-1^37,38^. All the above said points justified why ten natural compounds had very low negative values.

In this study, ten inhibitors had high affinity, and the docking score against NDM-1 and their binding revealed an exothermic pattern for Gibbs free energy and entropy changes. The value of MM-PBSA and MM-GBSA binding free energy was considerable for all compounds and also the results obtained from RMSD and RMSF plots exhibited that all ten compounds made strong and stable complexes with NDM-1. Thus, these compounds can compete with β-lactam antibiotics to bind with NDM-1 and survive them from hydrolyzes by this enzyme. These NPs are suggested as the potential lead to develop future drug candidates.

In the following, to estimate active sites' Hotspots, post-docking analyses were done. This part produced result which corroborates the finding of a great deal of the previous work in this field^39,40^. Hotspots of amino acids that could be employed more effectively with configured lead molecules against the binding site of NDM-1 were Ser217, Ser251, Asn220, Asp124, His250, Lys211, Ile35. Among them, Ile35 involved in the Van der Waals interactions to all complexes. Also, His189, Gly219, Asp124 and His122 significantly contributed to the hydrophobic interactions alongside Ile35. These amino acids acted an essential role in stabilizing the complex via hydrogen bonds or hydrophobic contacts. An insight into NDM-1 structures showed that a situated water molecule between Zn302 and Zn303 performed as a nucleophile during hydrolysis of the β-lactam ring of antibiotics. Analyses of the hydrolysis process by NDM also indicated prominent part served by the aforementioned residues in maintaining the overall structure and function of NDM-1.

Alignment on all types of NDM (NDM-1–NDM-30 Figure S3) showed that all the above-considered amino acids operated a pivotal role in binding with the active site and conserved in all types of NDM. Therefore, they can be used for the pharmacophore modeling and ligand discovery in future studies.

Our precise 3D conformational alignment over the tertiary structure of all metallo-β-lactamases and BLDB database alignment over these proteins' second structure proved that the above mentioned amino acids were conserved in all metallo-β-lactamases (most likely play a role in the catalysis reaction in the active site). Ser251, Asn220, Asp124, His250, His189, Gly219 and His122 residues were conserved in the B1 and B2 subclass and for all three subclasses Asp124, His250, His189 and His122 conserved. FMO quantum method was used in the present study to provide strong evidence for verifying the common crucial residues function in the interaction of beta-lactam with MBLs. FMO results completely approved the same pattern in the MBLs for PIEDA interaction with their ligand. This method indicated some His amino acids, Asp, Lys in the active site, had major tasks to interact in ligand. These outcomes exactly support initial conclusion of alignment, 3D conformational alignment, and pharmacophore modeling. In the PDB 5ZGE, ampicillin and for CPH, BJP biapenem were selected. In FMO calculation, this option shows that hot spot of different kinds of the MBLs had the same quantum behavior in exposure of different kinds of beta-lactam antibiotics. The same behavior was discovered through a similar contribution of the conserved vital residues in the different types of MBLs.

The discussed ligands based on these common pharmacophores and key residues may inhibit all MBLs, although more investigations and laboratory experiments are essential. It would be cost–benefit effective in drug design that one natural compound block broad types of resistant enzymes.

## Conclusion

The initial aim of the present study was to inhibit NDM-1 by natural compounds. Moreover, it was decided to investigate the active site residues and interactions of these amino acids to ligands. It was supposed that if our compounds were selected by this method, they could inhibit most of MBL.

Several 3D conformational alignments were applied on the subclasses of MBLs to find conservatory of the key residues playing a crucial role in the interaction with ligands. Thus, if our natural compounds can interact with these residues, they will possibly inhibit all types of MBLs. 3D alignment, investigation about the spatial location of crucial amino acids in the active site, and pharmacophore modeling can be employed as excellent and comprehensive drug design methods. To the best of our knowledge, this is the first study that employed FMO to analyze the structure of the MBLs' and their binding site and it is the first time in docking via Autodock Vina that some ligands had such low-grade affinity as if these natural compounds have evolved to block MBLs. In the Trott and Olson study^21^. approximate the lowest binding energy was about − 14 kcal/mol; nonetheless, the lowest affinity was − 24.3 kcal/mol in the present study. It was very lesser, previous results, so GRID MIF method was used as an excellent approach to show the cause of such a low affinity and docking accuracy.

Future studies about MBLs can benefit from our results, compounds and protocol to find the potential drug in order to inhibit MBLs. Our team selected natural products owing to their availability and natural compounds derived from this source were more cost-effective than small molecules. The ten chosen natural compounds can inhibit NDM-1 in computational methods, and based on our study, maybe they inhibit all or most of MBL classes. Among these ten NPs, NPC472454 was the best inhibitor for NDM-1 inhibition and it can be a candidate drug for MDR infections.

## Supplementary Information

Supplementary Information.

## Abbreviations

NDM-1: New Delhi metallo-beta-lactamase-1
MBL: Metallo-β-lactamases
MDR: Multidrug Resistance
RMSD: Root mean square deviation
RMSF: Root mean square fluctuation
NP: Natural product
