# Supplementary material for: Discovery of potential inhibitors against New Delhi metallo-β-lactamase-1 from natural compounds: in silico-based methods
Source: Sci Rep. 2021 Jan 27;11:2390. doi: 10.1038/s41598-021-82009-6 (PMC7841178; doi:10.1038/s41598-021-82009-6)
Supplement: Supplementary file 1 — Supplementary Information. [file 41598_2021_82009_MOESM1_ESM.docx]

**Discovery of Potential Inhibitors Against New Delhi Metallo-β-Lactamase-1 From Natural Compounds: In Silico-Based Methods**

Azhar Salari-jazi^1^ , Karim Mahnam^2^ , Parisa Sadeghi^1^, Mohamad Sadegh Damavandi^1^, Jamshid Faghri^1^

1. Department of Microbiology, School of Medicine, Isfahan University of Medical Sciences, Isfahan, Iran

2. Biology Department, Faculty of Sciences, Shehrekord University, Shahrekord, Iran


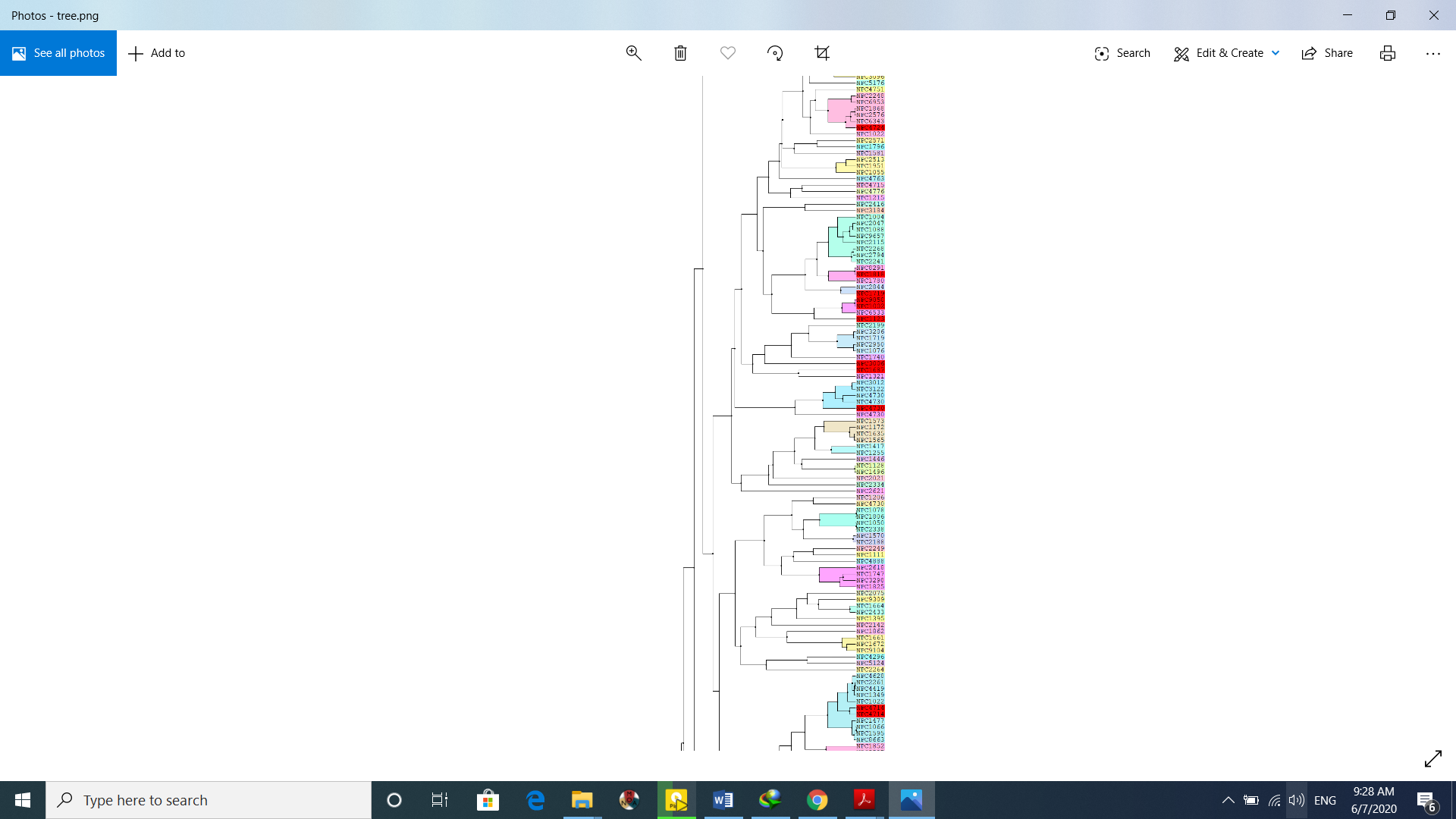


Figure 1. Hieratical clustering of the range of the our ten final compounds

Table 1. phisico-chemical properties

| **Name** | **# Acceptors** | **# Donors** | **# Rot. Bonds** | **MolWt** | **cLogP** | **TPSA** | **TPSA [Ertl]** |
| --- | --- | --- | --- | --- | --- | --- | --- |
| NPC18185 | 12 | 10 | 13 | 578.53 | 2.46 | 3.25 | 220.76 |
| NPC98583 | 18 | 14 | 23 | 882.74 | 3.96 | 3.62 | 354.28 |
| NPC100251 | 20 | 16 | 25 | 914.73 | 3.37 | 3.95 | 394.74 |
| NPC112380 | 18 | 13 | 21 | 868.71 | 3.14 | 3.7 | 351.12 |
| NPC120633 | 9 | 8 | 12 | 680.71 | 7.58 | 2.06 | 171.07 |
| NPC171932 | 10 | 8 | 12 | 562.53 | 3.21 | 2.83 | 189.53 |
| NPC300657 | 11 | 9 | 14 | 726.69 | 7.48 | 2.54 | 213.67 |
| NPC471403 | 15 | 10 | 13 | 814.75 | 3.57 | 2.64 | 248.45 |
| NPC471404 | 16 | 11 | 14 | 830.75 | 3.27 | 2.83 | 268.68 |
| NPC472454 | 13 | 9 | 12 | 590.49 | 2.07 | 3.61 | 234.67 |
| NPC473010 | 10 | 6 | 15 | 668.74 | 6.65 | 1.87 | 166.14 |


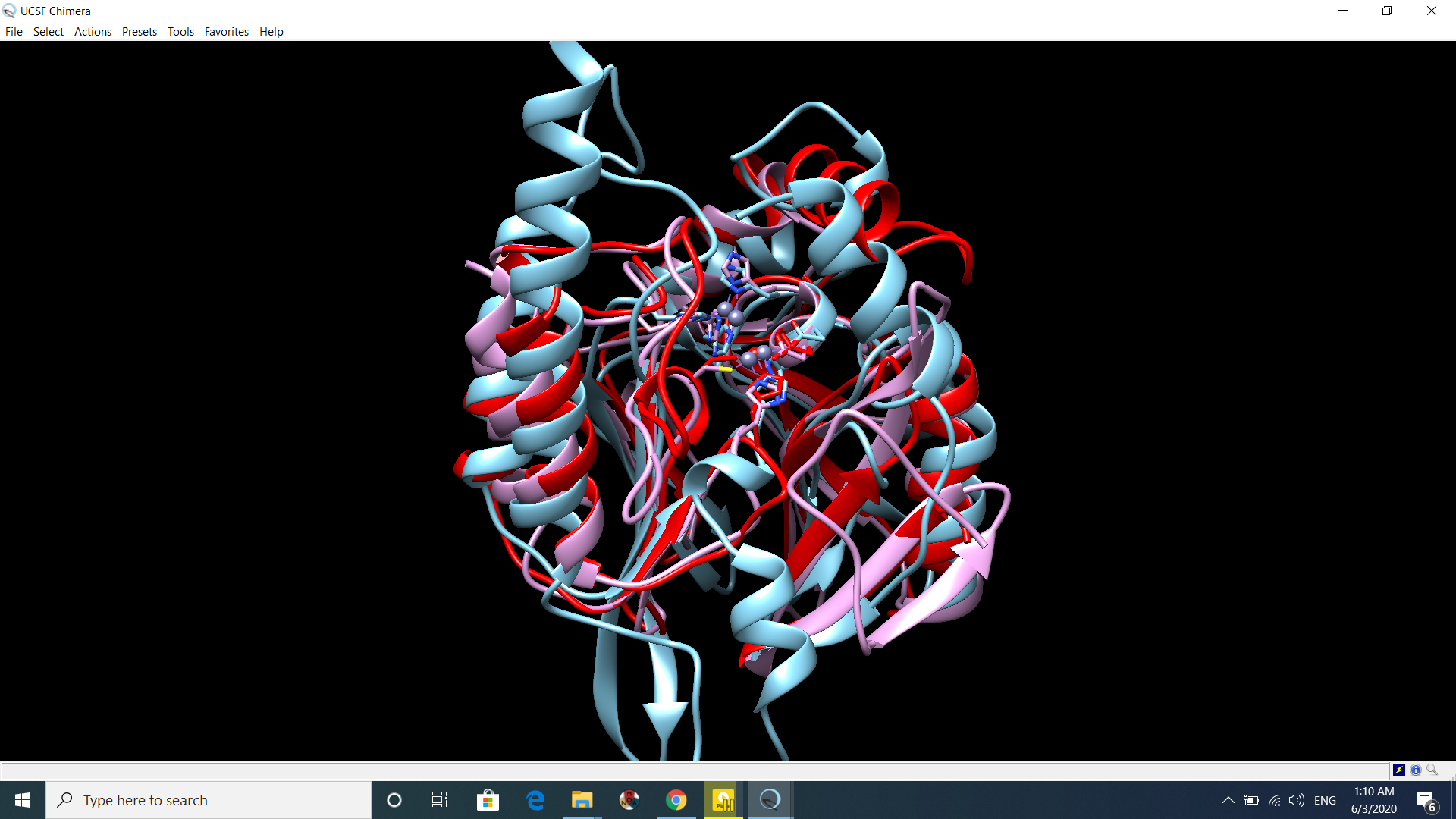


Figure 2. 3D alignment of the three class B1, B2 and B3 OF MBLs

| 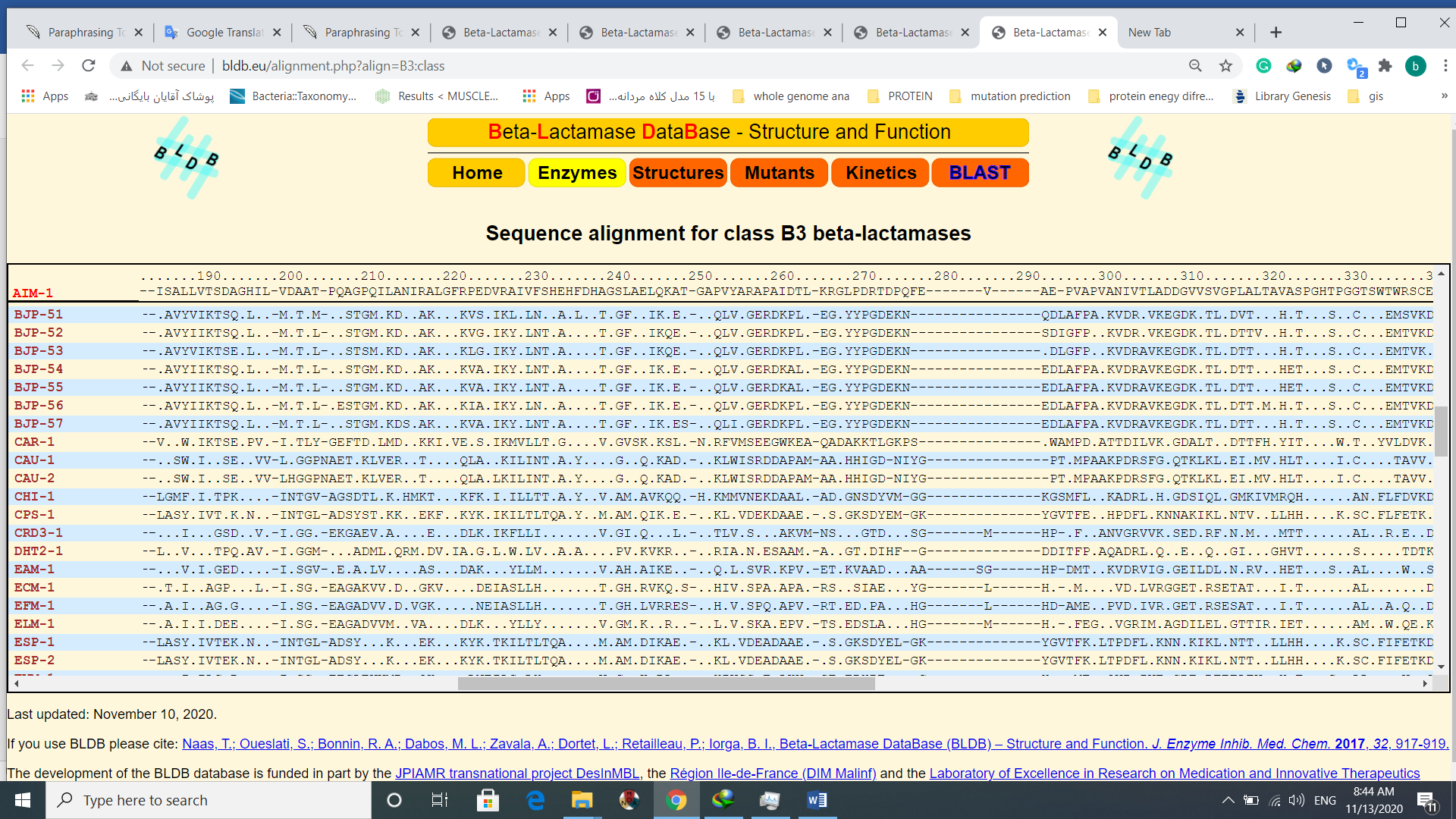 | 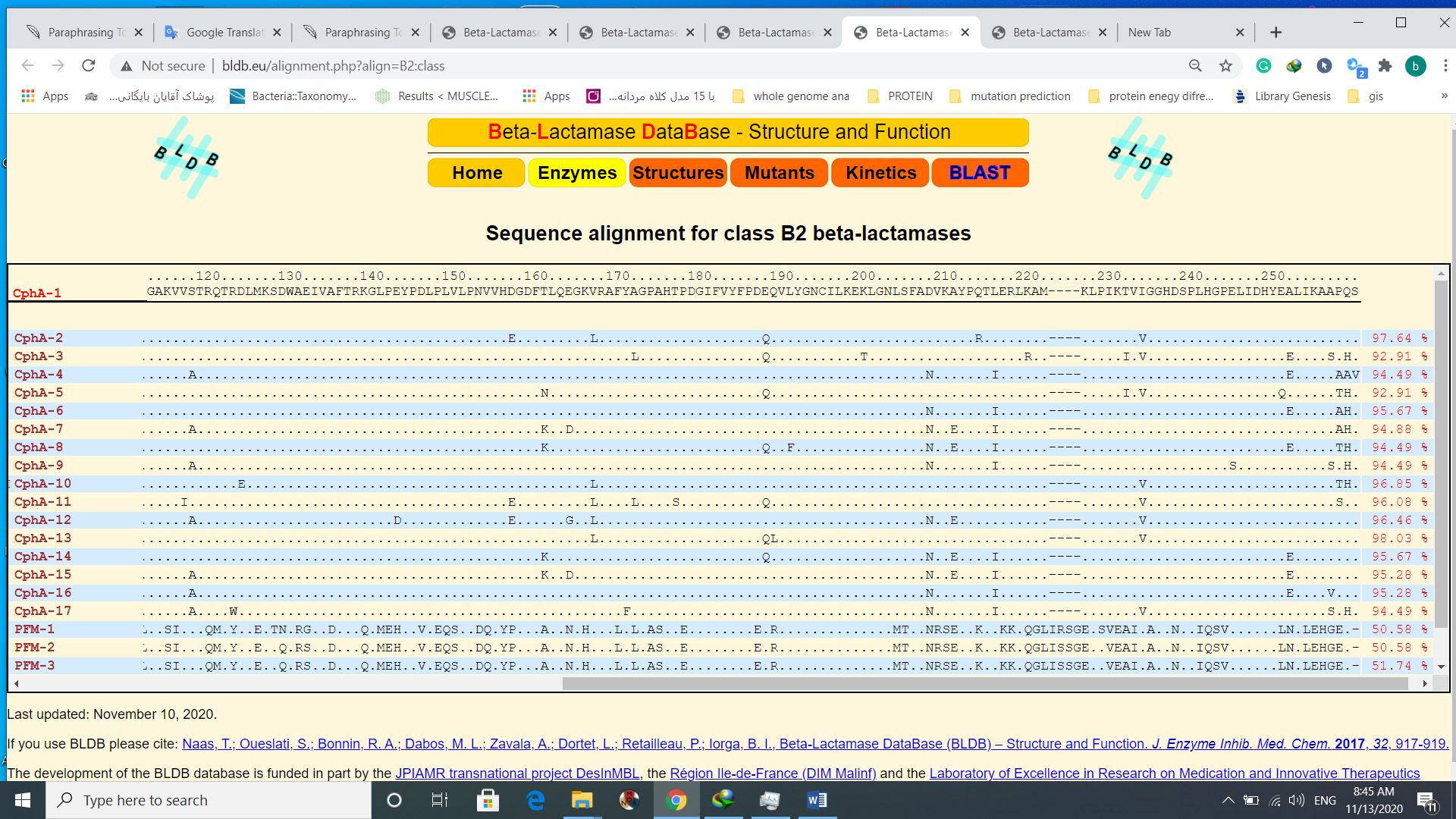 |
| --- | --- |
| 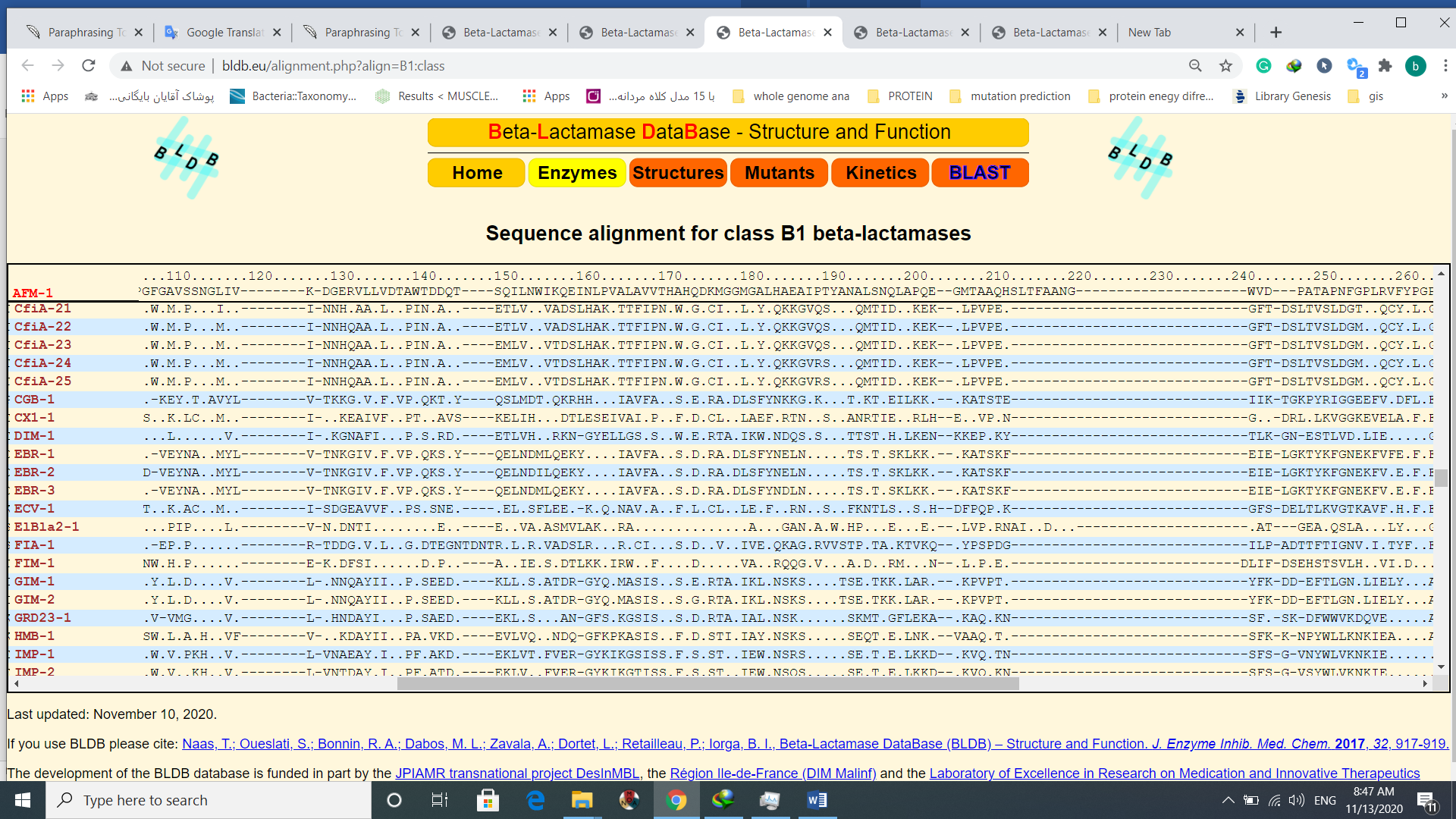 | 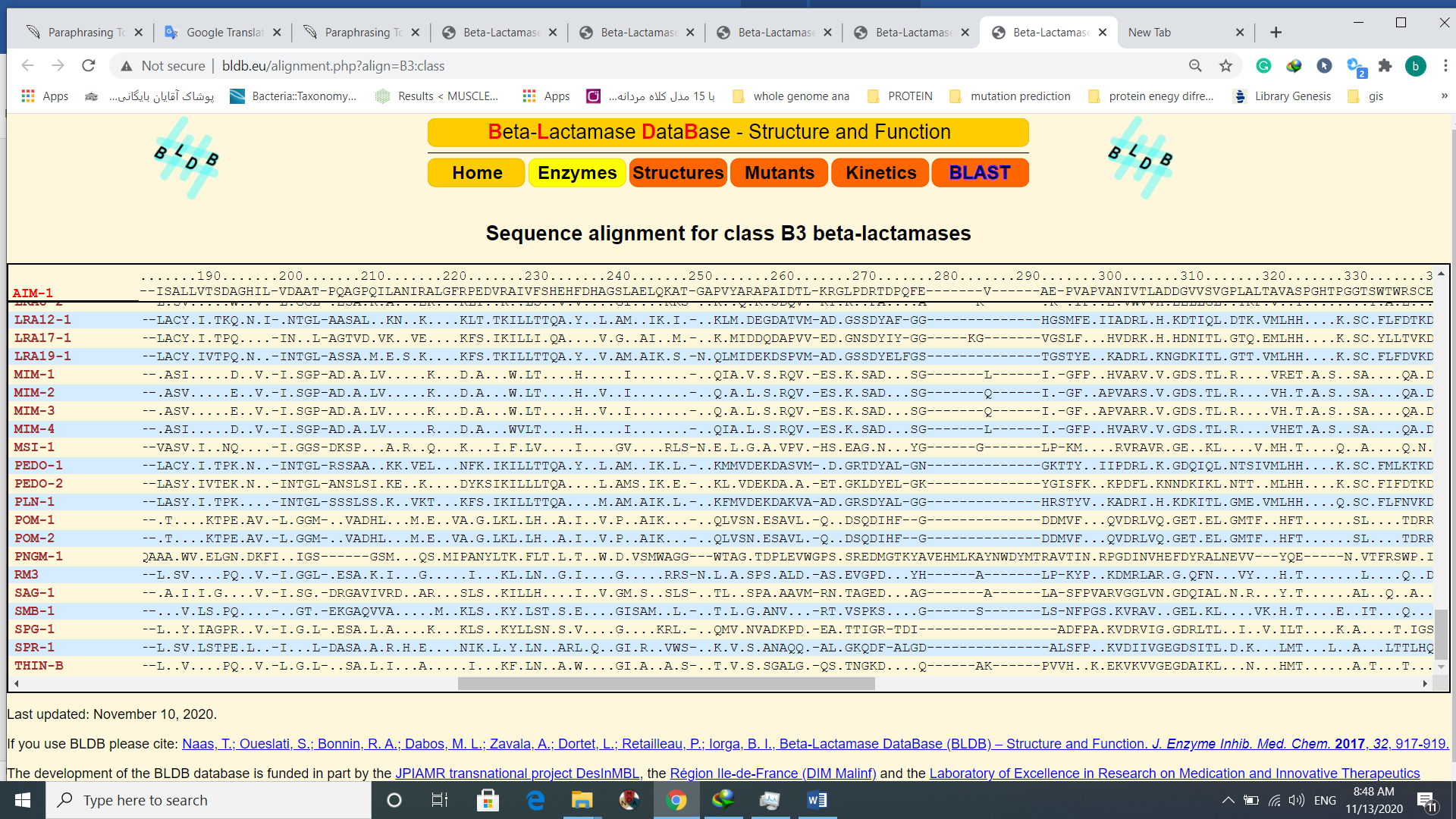 |
| 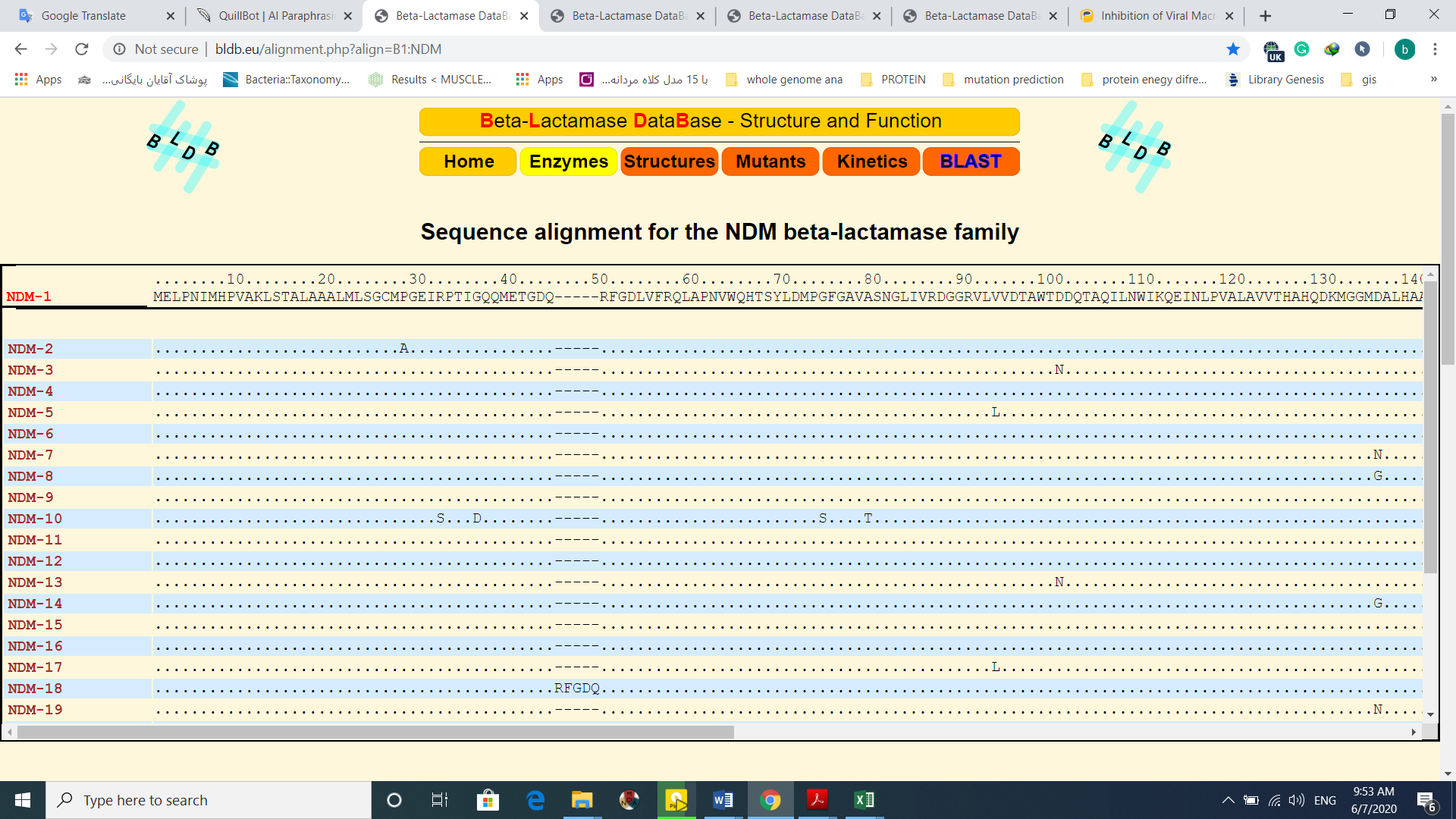 | 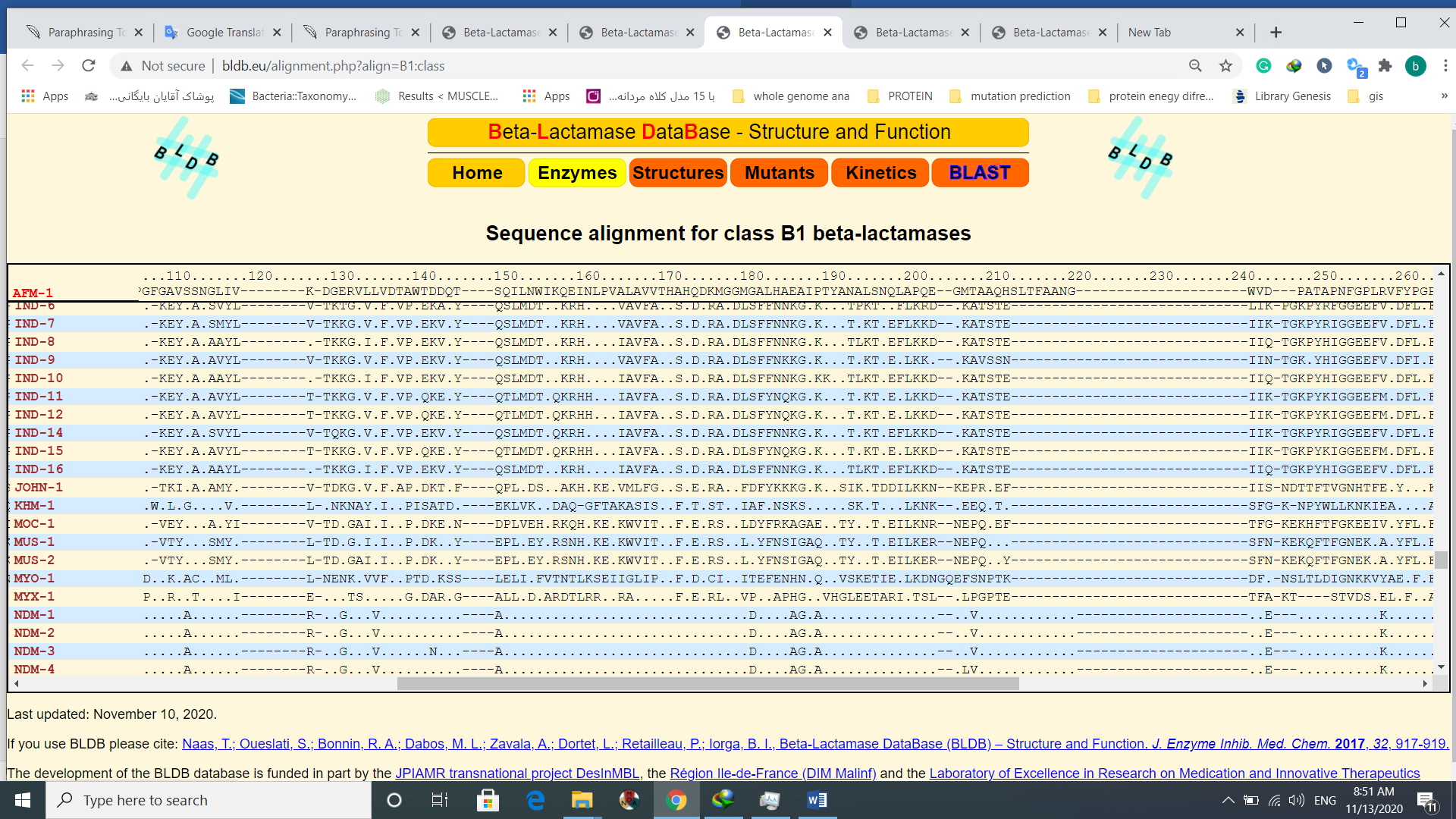 |

Figure 3. Alignment across all types of the MBLs


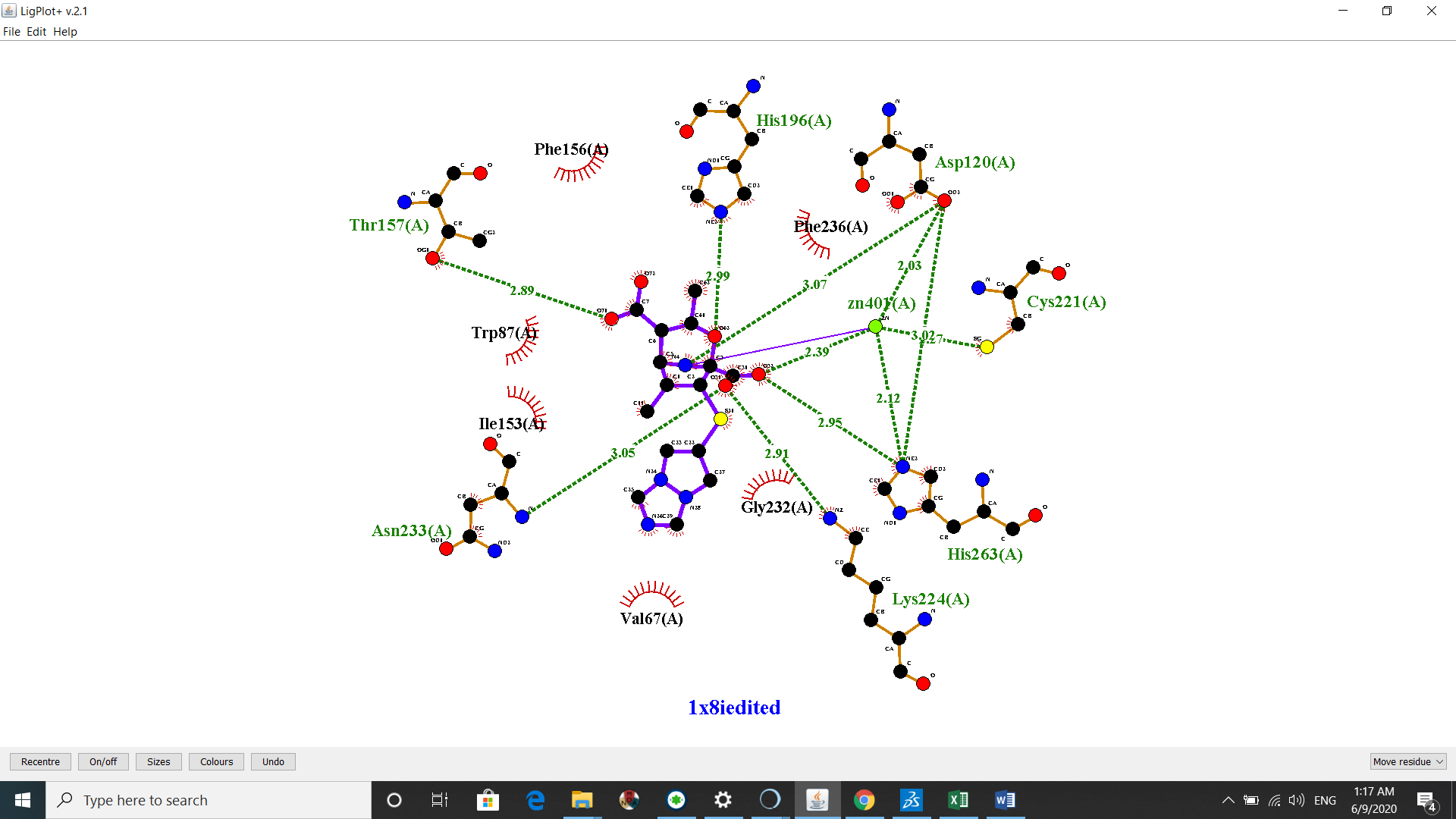


Figure 4. cphA-biapenem (B2). Interaction among three classes of the MBLS was similar to each other. These resemblance are comparable with Figure6 for BJP(B3) and NDM-1 (B1)


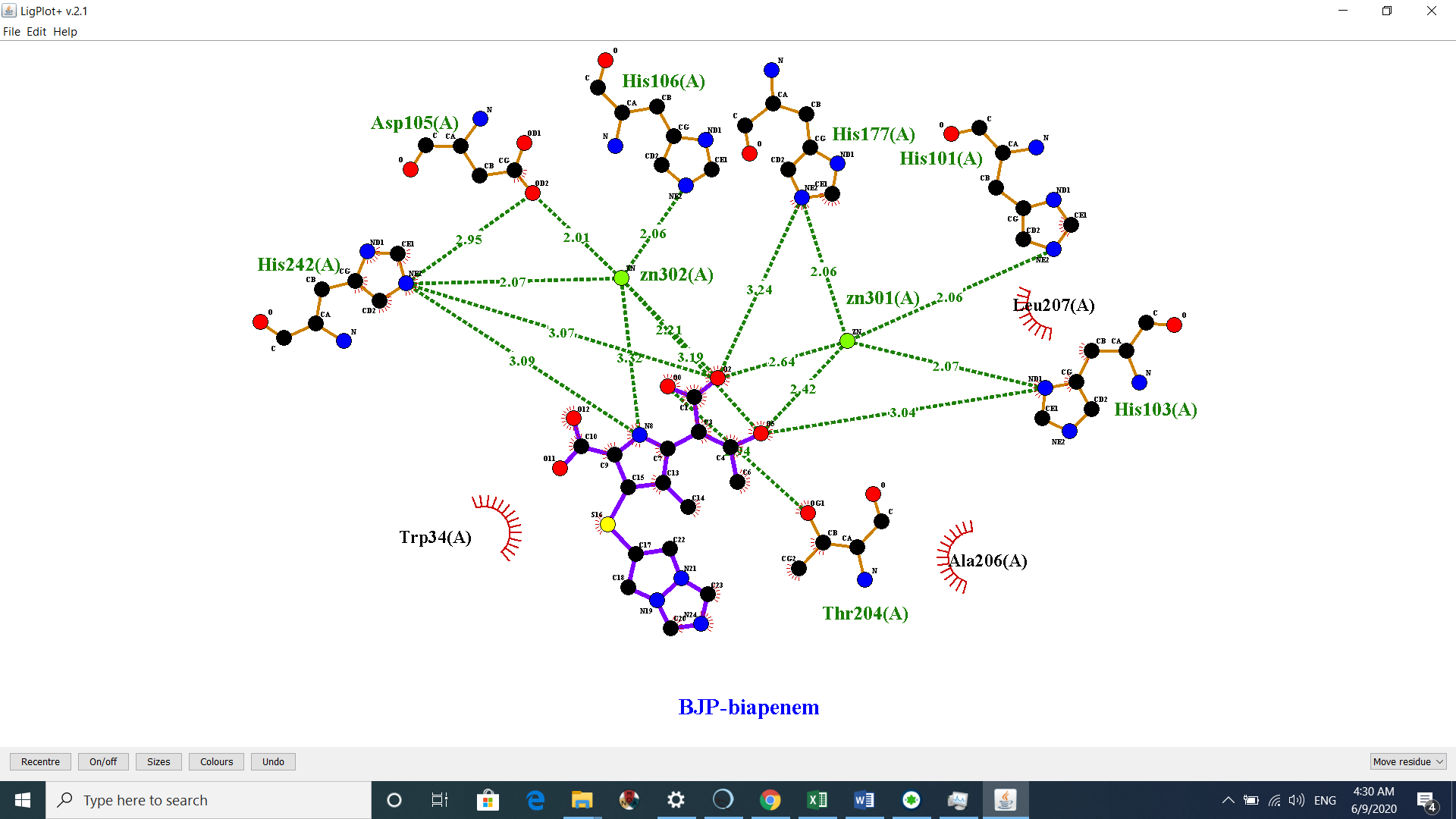


Figure 5. BJP-biapenem


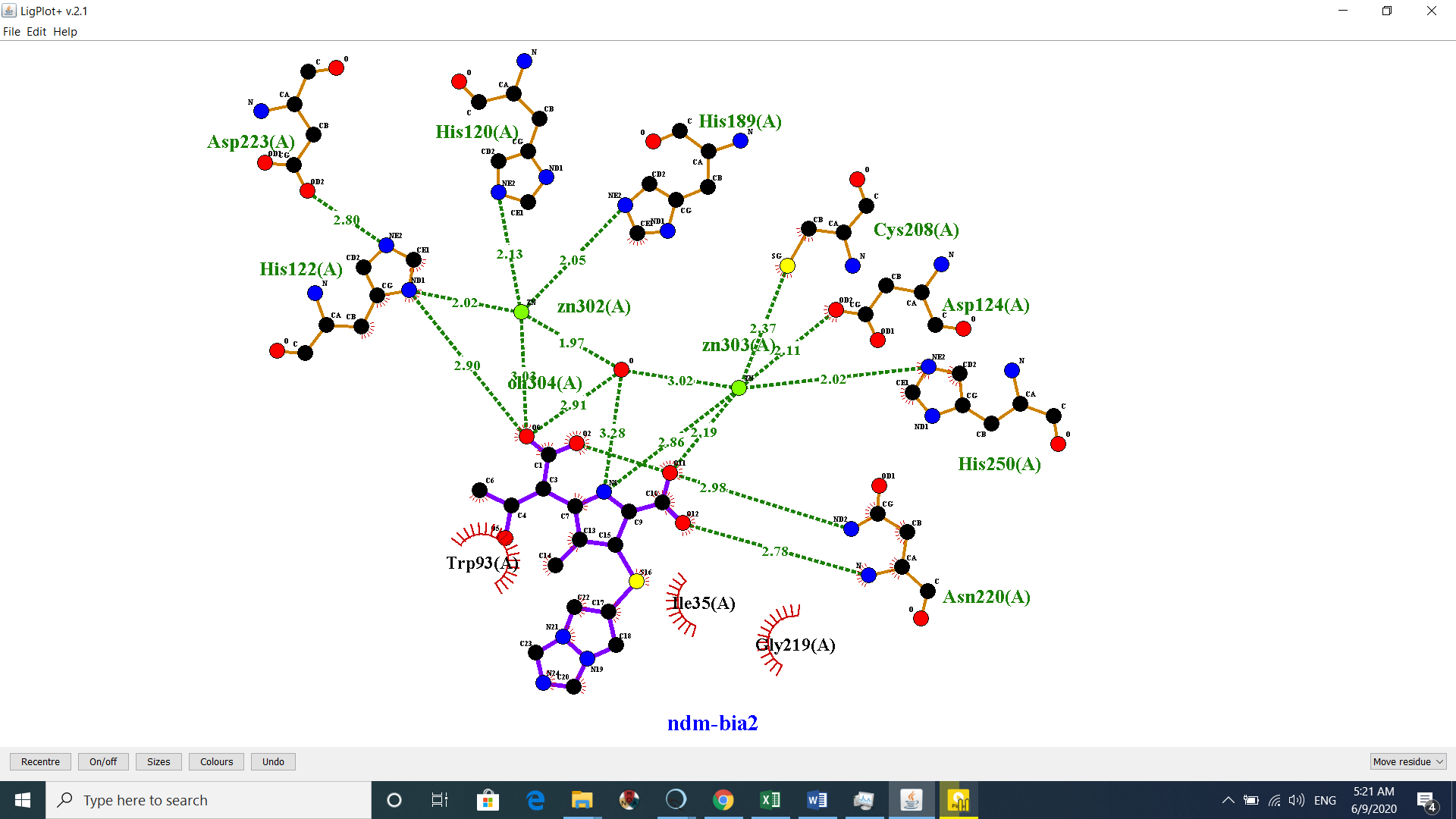


Figure 6. NDM-biapenem


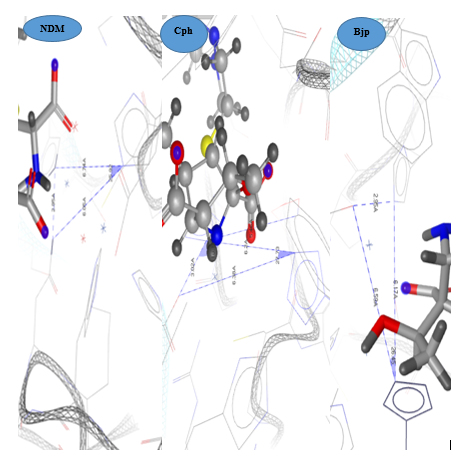


Figure 7. Distance and angle among the three example key residue key


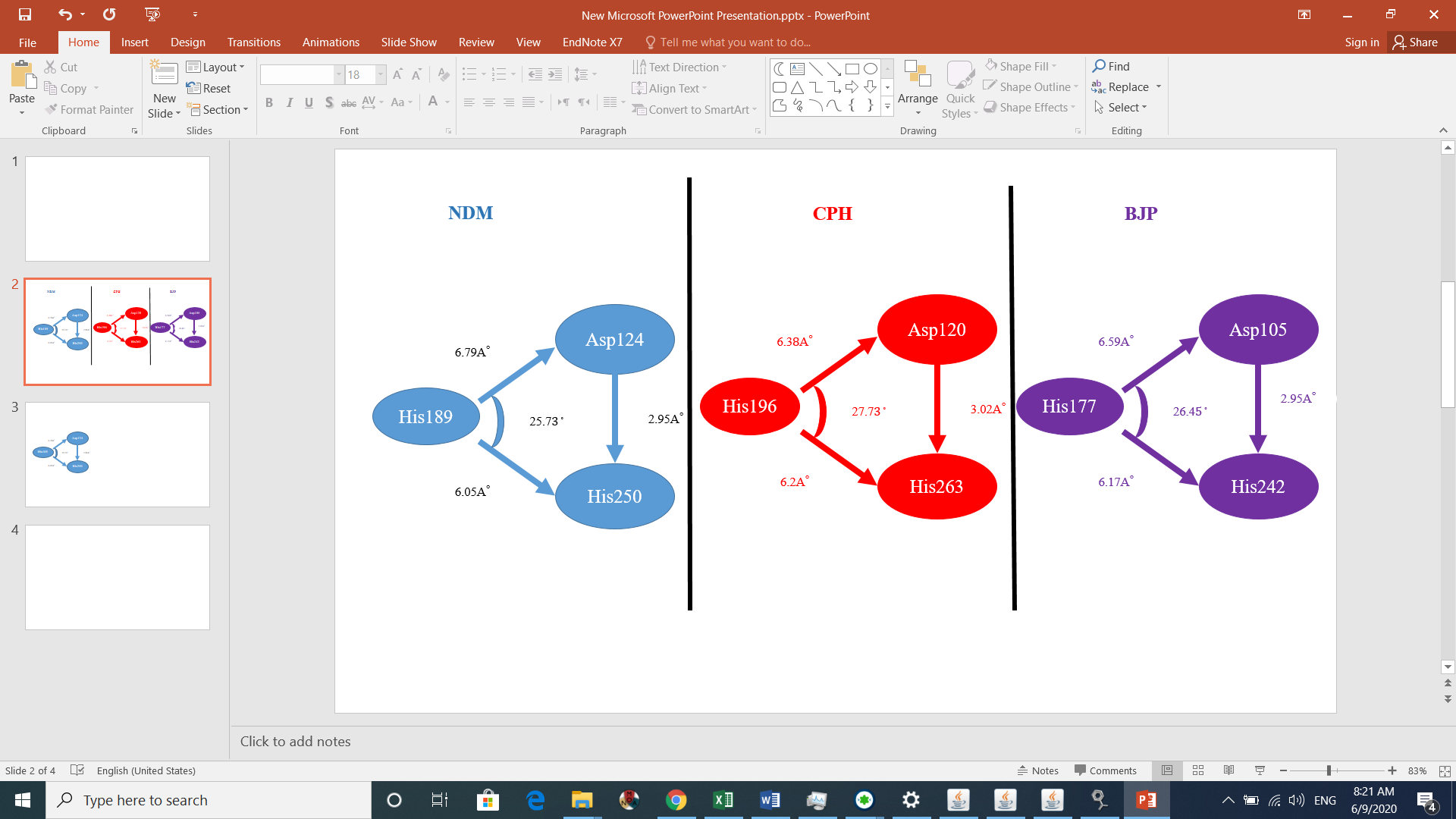


Figure 8. Distance and angle among the three examples key residues. This schematic picture showed high similarity of the coordination among analogous residue. Angels and distances between the key residue of B1, B2 and B3 classes are resemble.


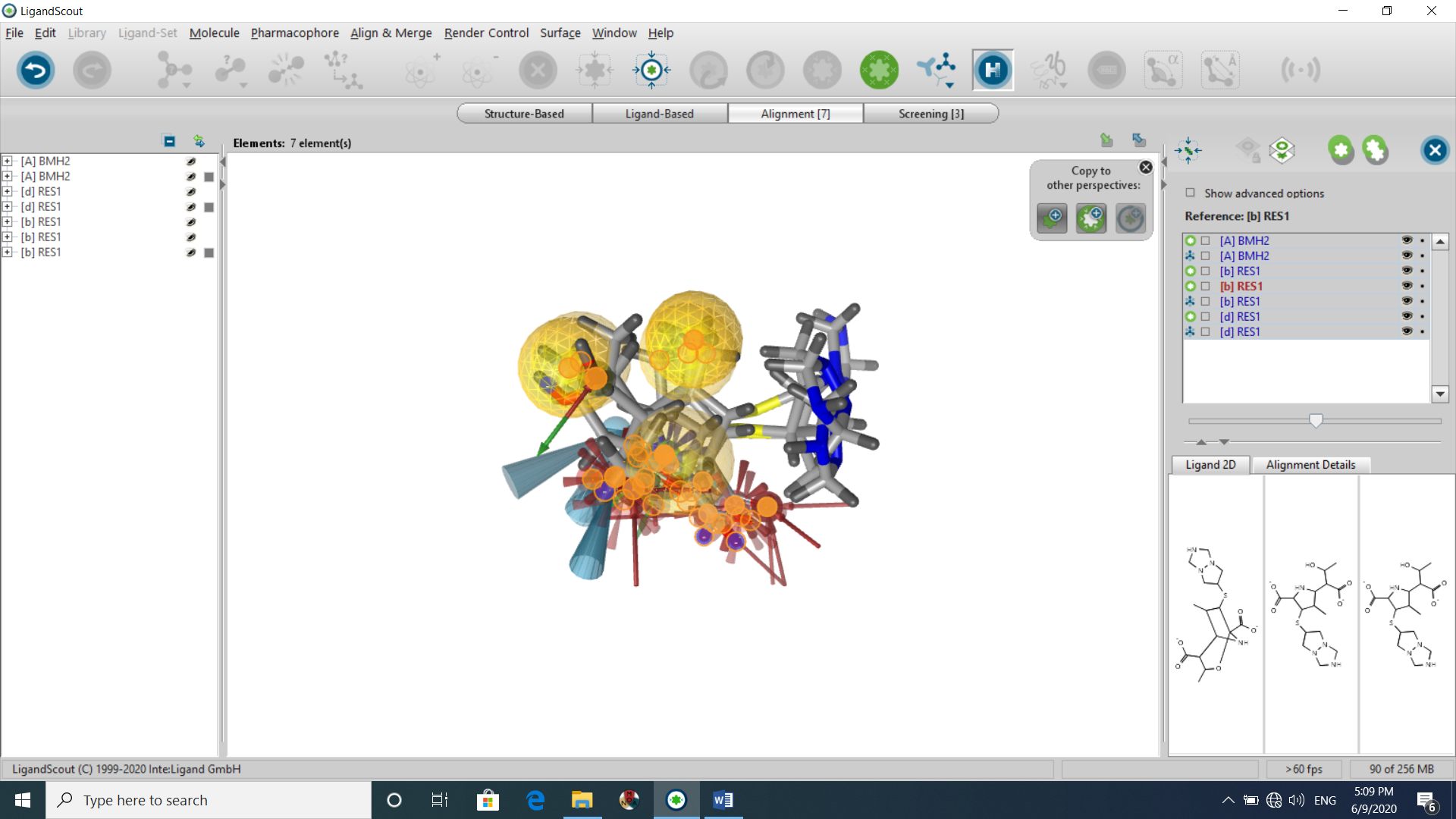


Figure 9. Matched biapenems which docked in the NDM, BJP and cphA. pharmacophores were similar among all of them.
